# ANG‐Modified Liposomes Coloaded With α‐Melittin and Resveratrol Induce Apoptosis and Pyroptosis in Glioblastoma Cells by Impeding Wnt/β‐Catenin Signaling

**DOI:** 10.1111/cns.70437

**Published:** 2025-05-21

**Authors:** Hai‐Qian Zhang, Yan Wang, Xiao Geng, Mingxin Dong, Ziwei Liu, Chengbiao Sun, Kaikai Yu, Wenwen Xin, Ye Xu, Na Xu, Wensen Liu

**Affiliations:** ^1^ Changchun Veterinary Research Institute Chinese Academy of Agricultural Science Changchun China; ^2^ The Second Hospital of Jilin University Changchun China; ^3^ State Key Laboratory of Pathogen and Biosecurity Institute of Microbiology and Epidemiology, AMMS Beijing China; ^4^ Basic College of Medicine Jilin Medical University Jilin China

**Keywords:** α‐Melittin, combination therapy, glioblastoma, pyroptosis, resveratrol, Wnt/β‐catenin signaling pathway

## Abstract

**Main Problem:**

Glioblastoma (GB) is one of the most prevalent and devastating types of brain cancer for which efficient treatments are currently lacking because of limitations such as antitumor efficacy, brain delivery, tumor selectivity, and drug resistance. A promising strategy to overcome these obstacles is developing anticancer agents that can be delivered to GB tissues to inhibit tumors with low toxicity to normal brain tissue.

**Methods:**

We developed liposomes encapsulating resveratrol (RES), a polyphenolic compound, and α‐melittin (α‐MEL), which is composed of melittin conjugated with an amphiphilic α‐helical peptide at its N‐terminus. RES‐, α‐MEL‐, and α‐MEL‐RES‐loaded liposomes (Lips) were modified with Angiopep‐2 (ANG). The effects of the above liposomes on GB cells were assessed, and the possible mechanisms were analyzed.

**Results:**

ANG‐modified α‐MEL‐RES‐Lips treatment facilitated the passage of these agents through the blood–brain barrier (BBB), increased tumor targeting, and significantly reduced α‐MEL‐associated hemolysis. The combined management of α‐MEL with RES impeded GB cell growth and prolonged the lifespan of GB tumor‐bearing model mice. α‐MEL‐RES‐Lips treatment triggered GB cell apoptosis and induced pyroptosis‐associated protein expressions of gasdermin‐D (GSDMD), gasdermin E (GSDME), cleaved caspase 3, and NLR family pyrin domain containing 3 (NLRP3), and inhibited epithelial–mesenchymal transition (EMT) by modulating the Wnt/β‐catenin signaling pathway.

**Conclusion:**

ANG‐modified α‐MEL‐RES‐Lips might be a potential nanosystem for GB therapy, and polyphenolic compounds combined with antimicrobial peptides may promote the induction of apoptosis, pyroptosis, and the apoptosis–pyroptosis switch in GB.

## Graphical abstract

ANG‐modified liposomes encapsulating α‐melittin and resveratrol increase their blood–brain barrier penetration and GB cell selectivity. The combined treatment of α‐melittin with resveratrol markedly facilitates apoptosis and pyroptosis, and promotes the apoptosis–pyroptosis switch in the GB cells via downregulating the Wnt/β‐catenin signaling.

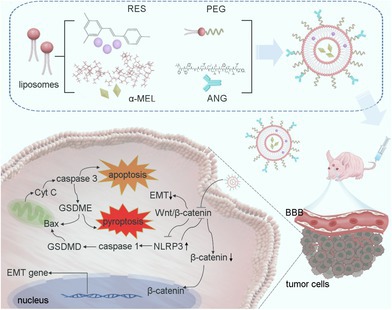

## 1 Introduction

Glioblastoma (GB), the most malignant type of glioma, originates in the brain and accounts for 80% of central nervous system malignancies. The median survival time of patients suffering from GB is only 1–1.5 years [1]. The characteristics of GB, such as its highly aggressive nature and unclear boundaries with normal tissues, lead to its inability to be completely removed by surgery. Moreover, the inability of chemotherapy drugs to effectively cross the blood–brain barrier to target GB is a challenge for GB treatment. Chemoresistance, radioresistance, and tumor recurrence also affect the survival and quality of life of patients with GB [2]. Thus, the identification of new therapeutic strategies is critical for GB treatment.

Melittin (C_131_H_229_N_39_O_31_) is a main component of bee venom. Melittin is composed of 26 amino acids (GIGAVLKVLTTGLPALISWIKRKRQQCONH2) [3] with two positive charges in the carboxy terminal region and four positive charges in the N‐terminal region. The amino terminal region, which includes residues 1–20, is hydrophobic, whereas the six amino acids in the melittin monomer are hydrophilic, strongly polar and cationic [4]. Melittin adopts an α‐helical secondary structure with a continuous hydrophobic surface on one side and a polar surface on the other side [5]. Melittin exhibits an affinity for anionic phospholipid membranes. At nanomolar concentrations, melittin can form transient pores on the cell membranes of eukaryotes and prokaryotes, and at micromolar concentrations, melittin can induce the formation of stable pores on the cell membrane [6]. There is an anionic phospholipid bilayer membrane on the tumor cells, and melittin can spontaneously bind to these anionic tumor cell membranes [7]. Melittin can also reorganize intramembrane cholesterol after binding to tumor cell membranes [8], which can effectively regulate membrane fluidity and permeability; thus, due to its inherent properties, melittin may not induce drug resistance [9]. Melittin exhibited anti‐glioma properties [10]. Melittin could enhance the oxidant activity of cisplatin and impede the growth of human GB cells [11]. On the other hand, melittin usually binds to cell membranes in a nonselective manner [12]. Thus, while inhibiting tumor cells, melittin can also damage healthy cells. Direct intravenous administration of melittin can cause serious toxic effects, such as hemolysis [13]. Melittin reduces the surface tension of the cell membrane and increases capillary permeability [14] and blood clotting time [15]. Injection of melittin (10 μg of melittin in a 50 μL volume of saline) could develop secondary heat hyperalgesia in human [16]. Intradermal injection of melittin (50 μg melittin in 50 μL 0.9% saline) induced spontaneous pain and neurogenic inflammation [17]. Furthermore, melittin is a small‐molecule peptide with a short half‐life and a high plasma clearance rate in the body, which makes melittin unsuitable for clinical applications due to its ease of degradation and metabolism [18]. Study has considered structurally modifying melittin, packaging it in a nanocarrier, and using it for combination therapy to address its side effects [19, 20]. Interestingly, after an amphipathic α‐helical peptide was linked to the N‐terminus of melittin via a GSG linker, generating α‐melittin, the melittin‐induced hemolytic effects were mitigated [21]. Jeong and colleagues designed a melittin and mertansine peptide–drug conjugate, which markedly increased the survival rates of melanoma model mice [22]. Additionally, combined treatment of melittin with epigallocatechin‐3‐gallate by fluorinated mitigated the hemolysis triggered by melittin while inhibiting the proliferation of and inducing apoptosis in human hepatocellular carcinoma cells in vitro and in vivo [23]. The combination of melittin with a natural medicine may reduce the biological limitations of melittin and increase its antitumor efficacy.

Resveratrol (5‐[(E)‐2‐(4‐hydroxyphenyl)ethenyl]benzene‐1,3‐diol) is a nonflavonoid polyphenolic compound derived from plants such as knotweed, cassia, and mulberry. In addition to its antioxidant properties, cardioprotective effects, and ability to regulate glucose hemostasis, resveratrol can prevent tumor cell proliferation, impede tumor metastasis, and trigger tumor cell apoptosis [24]. Resveratrol induces cell cycle arrest [25] and triggers apoptosis in glioma cell lines [26]. Resveratrol could reduce the clonogenic survival of glioma cells via inducing endoplasmic reticulum stress and oxidative stress [27]. Resveratrol can improve the efficacy of temozolomide in GB cells [28]. Pterostilbene, an analog of resveratrol, promotes mitochondrial apoptosis and induces S‐phase arrest in glioma cells [29]. However, the low water solubility [30], chemical instability [31], need for high doses, and rapid metabolism and elimination [32] of resveratrol limit its use. Kotta and colleagues developed a nanoemulsion formulation using a thermosensitive hydrogel to carry resveratrol, which effectively delivered resveratrol to suppress breast cancer cells [33]. Combining, derivatizing, and developing nanoparticle‐based delivery systems to encapsulate resveratrol may overcome its bioavailability limitations.

Angiopep‐2 (ANG), a member of the angiopep family of peptides, has considerable affinity for low‐density lipoprotein receptor‐related protein (LRP‐1), a transmembrane endocytic protein, and binds to its cytoplasmic tail [34]. Brain endothelial LRP1 is highly expressed and is considered a promising target receptor for delivering drugs across the BBB [35]. In addition, LRP1 is highly expressed in GB cells and brain metastases of breast, skin, and lung cancer [36]. ANG‐modified nanoparticles may enable antitumor agents to cross the BBB and target GB.

In the present study, we developed ANG‐modified liposomes to carry α‐melittin and resveratrol (ANG‐modified α‐MEL‐RES‐Lips) and assessed their effects on GB in vitro and in vivo. We showed that the ANG‐modified α‐MEL‐RES‐Lips ameliorated the hemolysis caused by MEL and inhibited the proliferation of the human GB cell lines T98G and HS683. ANG‐modified α‐MEL‐RES‐Lips presented synergistic effects on GB. ANG‐modified α‐MEL‐RES‐Lips treatment significantly triggered cell cycle arrest in the HS683 cell and induced apoptosis. The combination of α‐MEL with RES inhibited GB cell growth in a xenograft mouse model. Moreover, ANG‐modified α‐MEL‐RES‐Lips facilitated apoptosis and pyroptosis in GB cells and inhibited EMT by modulating the Wnt/β‐catenin signaling pathway.

## 2 Materials and Methods

### 2.1 Reagents

Melittin (M422487) was purchased from Aladdin Scientific Co. Ltd. α‐Melittin (DWFKAFYDKVAEKFKEAFGSGGIGAVLKVLTTGLPALISWIKRKRQQ‐NH2) (P230224‐HX1055653) was obtained from APeptide Co. Ltd. (China). Resveratrol (AB0774) was purchased from Chengdu Alfa Biotechnology Co. Ltd. (China). DSPE‐PEG2000 (1,2‐distearoyl‐sn‐glycero‐3‐phosphoethanolamine‐N‐[methoxy (polyethylene glycol)‐2000]), DSPE‐PEG2000‐COOH, DSPE‐PEG3400, DSPE‐PEG3400‐COOH, and NH2‐DSPE‐PEG3400 were obtained from Xian Ruixi Biological Technology Co. Ltd. (China). Soy lecithin and cholesterol were procured from Shanghai Macklin Biochemical Technology Co. Ltd. (China). 1‐Ethyl‐(3‐dimethylaminopropyl) carbodiimide hydrochloride (EDC·HCl) and N‐hydroxysuccinimide (NHS) were purchased from Shanghai Aladdin Biochemical Technology Co. Ltd. (China). Angiopep‐2 was purchased from Bankpeptide Co. Ltd. (China). Tween 80 was obtained from Beijing Biotopped Technology Co. Ltd. The antibodies and testing kits were present in Table S1.

### 2.2 Cells

Human GB cells (HS683 and T98G) were acquired from Boster Biological Technology Co. Ltd. (Wuhan, China). Human brain microvessel endothelial cells (hBMECs) were obtained from Wuhan Pricella Biotechnology Co. Ltd. (China). The cells were cultured in Dulbecco's modified Eagle's medium (DMEM) supplemented with 10% FBS (VivaCell Biotechnology) in a humidified atmosphere with 5% CO_2_ and 95% air at 37°C. HS683 cells were treated with lentiviruses expressing firefly luciferase and puromycin containing the EF1α promoter (Lenti‐EF1α‐Luc‐T2A‐Puro, Beyotime Biotechnology) and then screened with puromycin to generate HS683‐*luc* cells, which were used for tumor cell tracing in xenograft mice.

### 2.3 Animals

Male C57BL/6 mice aged 4 months and BALB/c nude mice aged 4–6 weeks were obtained from HFKBIO Co. Ltd. (Beijing, China) and housed under specific pathogen‐free conditions. All animal procedures were performed according to Chinese regulations involving animal protection and were approved by the Animal Ethics Committee of Jilin Medical University (2020‐KJT064) on September 3, 2020.

### 2.4 Preparation of ANG‐Modified α‐MEL‐RES‐Lips

ANG‐modified α‐MEL‐RES‐Lips was prepared by thin‐film hydration. A total of 330 mg of soy lecithin and 66 mg of cholesterol were dissolved in 5 mL of anhydrous ethanol. Then, 30 mg of resveratrol and 20 μL of Tween 80 were added to each solution, and the mixtures were placed in an ultrasonic water bath to allow dissolution of the reagents. After being transferred to an eggplant‐shaped flask, the solutions were placed on a rotary evaporator to remove the ethanol (vacuum, 35°C, 90 rpm). When a thin film formed, the samples were hydrated with 20 mL of physiological saline (PBS, 0.01 M). The obtained samples were sonicated for 10 min (300 W, 5‐s intervals), and the DSPE‐PEG inclusion complex (composed of soy lecithin, DSPE‐PEG2000, DSPE‐PEG2000‐COOH, DSPE‐PEG3400, and DSPE‐PEG3400‐COOH at a molar ratio of 95:2.7:0.3:1.8:0.2) was added. Ten milliliters of the above solutions were added to 5 mg of α‐MEL. Under continuous magnetic stirring, EDC·HCl and NHS were added at a molar ratio of soy lecithin to EDC·HCl and NHS of 10:1:1. After the reagents were thoroughly dissolved in the dark, Angiopep‐2 was added to the obtained samples at a molar ratio of Angiopep‐2 to soy lecithin of 1:30. ANG‐modified α‐MEL‐RES‐Lips were collected using a 0.22 μm pore size microporous membrane.

### 2.5 Characterization of the ANG‐Modified α‐MEL‐RES‐Lips

The chemical structure of the liposomes was assessed by Fourier transform infrared (FT‐IR) spectroscopy (BRUKE, VERTEX70). After freeze drying, the samples were mixed with potassium bromide (KBr) at ratios ranging from 1 to 20 and ground under an infrared lamp. Each sample was then pressed into a thin transparent pellet at 12 N and examined in the wavelength range of 4000 to 400 cm^−1^ at room temperature.

The morphology and diameter of the liposomes were evaluated using a transmission electron microscopy (TEM) (JEM‐2100; Nikon). The particle size and zeta potential of the liposomes were analyzed using laser diffraction or Doppler velocimetry (25°C) with a Zetasizer instrument (Nano ZS90, Malvern Instruments Ltd.). The encapsulation efficiency (EE) and loading efficiency (LE) were determined in accordance with previous methods [37]. The EE and LE were calculated as follows: EE (%) = $\frac{\text{Total amount of drug}-\text{amount of drug in the supernatant}}{\text{Total amount of drug}}$ × 100 and LE (%) = $\frac{\text{Total amount of drug}-\text{amount of drug in the supernatant}}{\text{Total amount of liposomes}}$ × 100. Drug release in vitro was investigated by dynamic dialysis. Two milligrams of ANG‐modified α‐MEL‐RES‐Lips was transferred to a dialysis bag (molecular weight cutoff: 14000 Da) and then placed into an oscillator containing 0.01 M PBS (pH 6.8 or pH 7.4). The amount of RES in each sample obtained at the indicated times (1, 2, 4, 6, 8, 10, 24, 48, 72 and 96 h) was determined spectrophotometrically at 306 nm [38]. α‐MEL concentrations in α‐MEL‐RES‐Lips without ANG modification were assessed by using the MicroBCA Protein Quantification Kit [39].

### 2.6 Hemolysis Assessment

Fresh blood cells from C57BL/6 mice were collected in anticoagulant tubes containing sodium heparin. The red blood cells were separated by centrifugation at 1500 rpm for 5 min and then diluted to 1.0 × 10^6^/mL^21^ in 0.01 M PBS. Three hundred microliters of the red blood cell suspension were treated with various concentrations (5, 10, 20, 50, 100, 200 and 500 μg/mL; each at the same volume) of free melittin, melittin‐Lips, α‐melittin, and α‐melittin‐Lips for 1 h (37°C). Then, the absorbance of the supernatant from each sample was measured at 550 nm using a microplate reader. Red blood cells incubated with 1% Triton X‐100 were used as positive controls.

### 2.7 Delivery Efficiency

Transwell assays were conducted to assess the ability of the liposomes to deliver RES and α‐melittin across the BBB. hBMECs were inoculated in the upper transwell chamber. When T98G or HS683 cells reached confluence, they were seeded in the bottom transwell chamber. The transendothelial electrical resistance (TEER) was measured daily. After a tightly confluent monolayer formed, the hBMECs were treated with a DMSO‐dissolved coumarin‐6 (Cou6) suspension (6 μM) or Cou6‐loaded Lips for 24 h. The T98G cells or HS683 in the bottom chamber were then collected and subjected to fluorescence signals analysis.

The distribution of the ANG‐modified liposomes in the mice was evaluated using an in vivo imaging system (IVIS Spectrum, Perkin‐Elmer, Massachusetts). The liposomes above were labeled with DiR (1,1′‐dioctadecyl‐3,3,3′,3′‐tetramethylindotricarbocyanine iodide, Absin Bioscience Inc., abs45153692). Three milligrams of DiR were added to 10 mL of the indicated liposomes for 4 h of incubation in the dark. After filtration, the DiR‐labeled samples were injected into the nude mice via the tail vein at a dose of 3 mg/kg. The in vivo fluorescence distribution was monitored at 2, 12, 24, and 36 h at an excitation wavelength of 748 nm and an emission wavelength of 780 nm. The DiR fluorescence intensity was quantified using Image J software.

### 2.8 In Vitro Growth Inhibition Assays

The effects of α‐MEL‐RES‐Lips on the growth and survival of T98G and HS683 cells were evaluated. Cells were plated in 96‐well microtiter plates (1 × 10^4^ per well). When they reached 70% confluence, the cells were cultured in serum‐free medium for 2 h. The cells were then incubated with RES‐Lips, α‐MEL‐Lips, or α‐MEL‐RES‐Lips for 24 h and then adding CCK‐8 reagent (GK10001, GlpBio, California) to the medium. The absorbance was measured at 450 nm to assess the number of live cells. Cell viability was calculated as follows: cell viability (%) = (average absorbance of the treated group—absorbance of the blank well)/(average absorbance of the control group—absorbance of the blank well) × 100%. The levels of lactic dehydrogenase (LDH) were determined using an LDH in vitro toxicology assay kit (Sigma–Aldrich) according to the manufacturer's instructions. The release of LDH into the culture medium was used as an indicator of the integrity of the cell membrane. LDH release has been considered one of reliable indicators of cell membrane integrity. It is noted that LDH is also influenced by intracellular metabolic shifts. In the present study, in order to control the potential confounding factors, the working solution of LDH analysis should be prepared and used immediately, and the experimental procedure was conducted to avoid light. The samples should be measured in time after collection without freezing. The cells were transferred to serum‐free medium 2 h before drug treatments to prevent the interference of lactate dehydrogenase in serum.

The effects of α‐MEL‐RES‐Lips treatment on the cell cycle were also assessed. Briefly, the cells were incubated in propidium iodide (PI) (1 mg/mL) at 4°C in the dark for 30 min. The number of stained cells was calculated via flow cytometry (FCM).

To evaluate the concentrations of the indicated treatments that triggered apoptosis, cells were first collected and treated with RNase. After being washed, the cells (at a concentration of 5 × 10^5^/mL) were fixed with 75% ethanol for 1 h (4°C) and then resuspended in binding buffer from an Annexin V‐fluorescein isothiocyanate (Annexin V‐FITC)/PI kit (Cell Signaling Technology Inc.). Five microliters of Annexin V‐FITC and 5 μL of PI were then added to the above samples, which were incubated for 20 min in the dark. The samples were then subjected to FCM.

### 2.9 Safety of the ANG‐Modified α‐MEL‐RES‐Lips

To evaluate the toxicity of ANG‐modified α‐MEL‐RES‐Lips in vivo, male C57BL/6 mice (4 months old) were divided into five groups and received one of the following via intraperitoneal injection: 0.9% physiological saline, ANG‐RES‐Lips, ANG‐α‐MEL‐Lips, ANG‐α‐MEL‐RES‐Lips, or vehicle control without encapsulating α‐MEL or RES. The mice were treated as indicated once per day for 14 days. The general health and body weights of the mice were monitored. On the 15th day, the mice were transcardially perfused with physiological saline, and the heart, liver, spleen, lung, kidney, and brain were collected. The obtained tissues were fixed, dehydrated, and sliced into 10 μm thick sections using a freezing microtome. The tissue sections were stained with hematoxylin and eosin (H&E). The physiological examination of blood cells and biochemical test of serum in the mice were performed using Catalyst One Chemistry Analyzer (IDEXX Laboratories Co. Ltd. Shanghai, China).

### 2.10 Antitumor Evaluation In Vivo

To establish mouse models bearing xenograft tumors, stereotactic injection was performed in female nude mice at the age of 5 weeks old. HS683‐luc cells were suspended in 0.01 M PBS to a concentration of 5 × 10^8^/mL. A total of 10 μL of the cell suspension was injected into the right frontal lobe 1.8 mm lateral, 1.0 mm anterior and 3.0 mm ventral from the bregma. From days 10 to 30 of postimplantation, the mice with established HS683‐luc GBM were intravenously managed with ANG‐modified RES‐Lips (RES dose: 15 mg/kg), ANG‐modified α‐MEL‐Lips (α‐MEL dose: 5 mg/kg), ANG‐modified α‐MEL‐RES‐Lips or physiological saline once every other day via the tail vein. The mice were imaged on days 10, 14, 18, 22, 24 and 28 to monitor the luciferase intensity of the tumors in the mice brains using an IVIS spectrum. From days 31 to 32, behavioral testing were performed to evaluate the spontaneous motor function and exploratory behavior by open field test. The sample size was determined by the power analysis approach named “resource equation approach” [40] and analysis of variance (ANOVA) [41] as previously described. The sample size was calculated as follow: *n* = $\frac{\mathrm{DF}}{k}$ + 1. “DF” means degrees of freedom, and the acceptable range of which is 10 to 20. “*k*” means the number of groups. “*n*” means the number of mice *per* group.

### 2.11 Open Field Test

The open field test (OFT) was performed to evaluate the motor function and exploratory behavior of the mice. Mice were firstly allowed to adapt to the new environment by placed in the center arena for 5 min. The next day, the exploratory behavior was monitored. The total distance and the number of times that the mice entered into the center of the arena were recorded and calculated by SMART v.3.0 software (Harvard Apparatus, British).

### 2.12 Immunostaining

For immunostaining analysis of protein expression in vitro and in vivo, cells or tissue sections were fixed with 4% paraformaldehyde and blocked with normal donkey serum (1:20; Jackson ImmunoResearch Laboratory, USA) for 30 min at room temperature. The samples were incubated with primary antibodies for 12 h at 4°C. After thorough rinsing, the samples were treated with a fluorescently labeled secondary antibody (goat anti‐mouse or anti‐rabbit IgG) for 2 h at room temperature. The nuclear chromatin was stained with 4ʹ,6‐diamidino‐2‐phenylindole (DAPI). The immunostaining results were observed with an Olympus microscope.

### 2.13 Nuclear and Cytoplasmic Isolation

The cultured cells were collected and homogenized in ten volumes of precold hypotonic buffer containing 10 mM 4‐(2‐hydroxyerhyl)piperazine‐1‐erhanesulfonic acid (HEPES), 10 mM KCl, 2 mM MgCl_2_, 1 mM dithiothreitol, 0.1 mM ethylene diamine tetraacetic acid (EDTA) and 0.1 mM phenylmethylsulfonyl‐fluoride (PMSF) from Nuclear Extraction Kit (Abcam). The samples were vigorously vortexed for 10 s to ensure that the cell precipitate was completely suspended and dispersed. After incubating with the hypotonic buffer for 10 min at 4°C, the samples were centrifuged at 14,000 *g* for 5 min. The supernatant was obtained as the cytosolic fraction. The pellets were treated with the hypotonic buffer above plus 40 μL 10% Nonidet (NP‐40) and then centrifuged. The precipitates were incubated in 50 μL of pre‐cold extraction buffer containing 20% glycerol, 50 mM HEPES, 50 mM KCl, 300 mM NaCl, 0.1 mM EDTA, (0.5 mM dithiothreitol and 0.1 mM PMSF) for 30 min with vortex for 15 s every 2 min. The specimens were centrifuged at 14,000 *g* for 10 min. The supernatant was collected as the nuclear proteins.

### 2.14 Western Blotting

Total protein from whole‐cell lysates was collected with RIPA buffer (pH 8.0) supplemented with a protein protease inhibitor cocktail (Sigma–Aldrich). Protein levels were measured via bicinchoninic acid assays. Thirty micrograms of total protein from each sample were separated via 8%–12% sodium dodecyl sulfate–polyacrylamide gel electrophoresis. After being transferred to polyvinylidene fluoride membranes (Millipore, Billerica, MA), the proteins were probed with primary antibodies and treated with HRP‐conjugated secondary antibodies of the appropriate species. The immunoblots were visualized using an enhanced chemiluminescence kit. The intensity of the immunoreactive bands was quantified using Image J software.

### 2.15 Assessment of Oxidative Redox

Malondialdehyde (MDA) contents were measured using a MDA Detection Kit (Nanjing Jiancheng Bioengineering Institute, Nanjing, Jiangsu, China) in accordance with the manufacturer's instructions. The contents of reduced glutathione (GSH) and oxidative glutathione (GSSG) were determined using a colorimetric assay kits (Beyotime Institute of biotechnology, Jiangsu, China). The products were read at 412 nm.

### 2.16 Statistical Analysis

The data are presented as the means ± standard deviation of the means (SD). A value of *p* < 0.05 was considered to indicate statistical significance.

## 3 Results

### 3.1 The α‐MEL‐RES‐Loaded Lips Formed a Uniform Dispersion, Increased the Stability of RES, and Inhibited Hemolysis Induced by MEL

**FIGURE 1:**
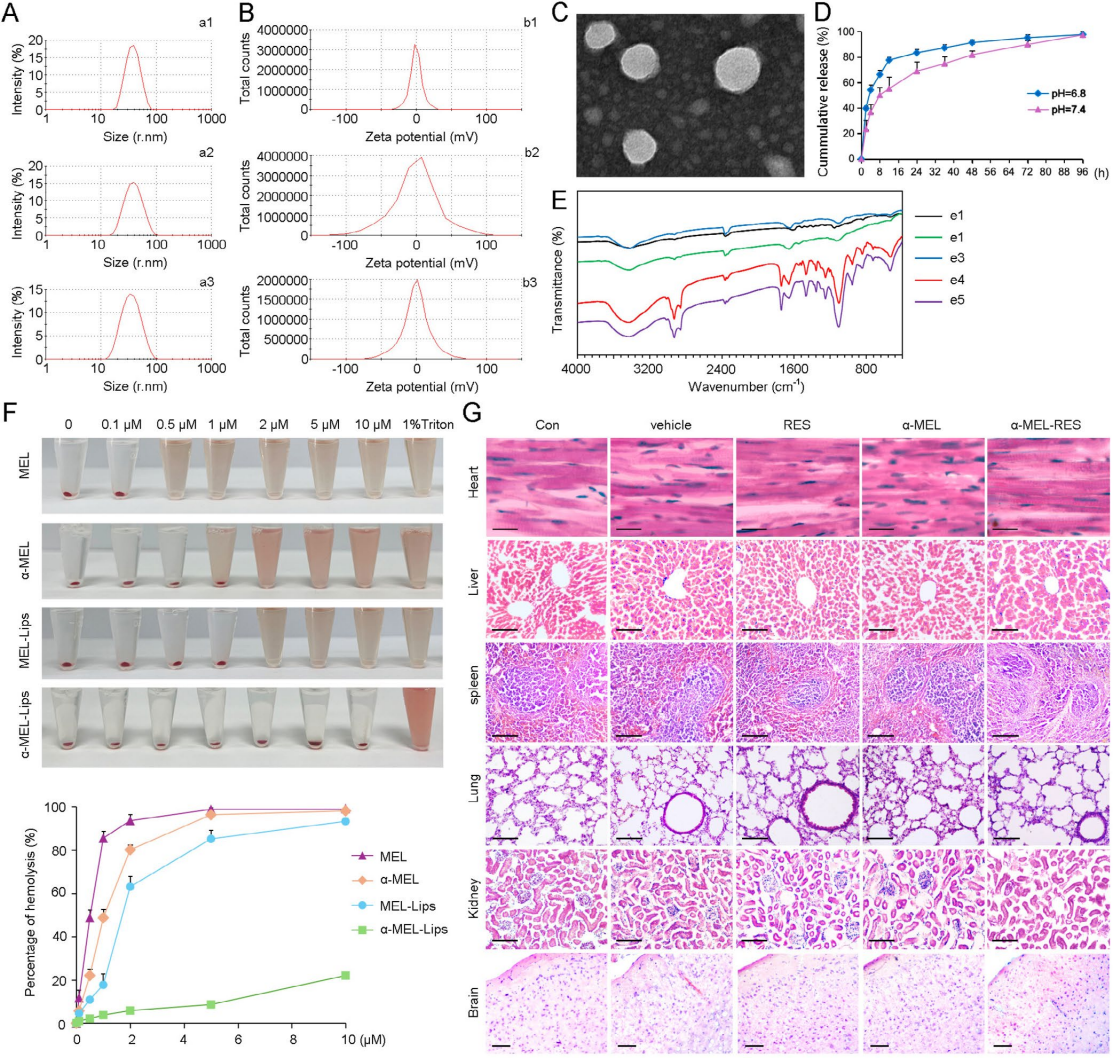
Characterization and safety assessment of RES‐, α‐MEL‐ and α‐MEL‐RES‐liposomes. (A) Size (radius) distributions of liposomes (Lips, a1), RES‐loaded Lips (a2) and α‐MEL‐RES‐Lips (a3) were measured at room temperature. (B) Zeta potential analysis indicated the charge conditions of Lips (b1), RES‐loaded Lips (b2) and α‐MEL‐RES‐Lips (b3). (C) TEM assays showing the morphology of Lips. Scale bar: 50 nm. (D) RES release from Lips in PBS were monitored at pH 7.4 and 6.8. (E) FT‐IR spectroscopy assays indicate the transmittance of RES (e1), α‐MEL (e2), ANG (e3), ANG‐modified α‐MEL‐RES‐Lips (e4) and the mixture of RES, ANG and α‐MEL‐RES‐Lips without EDC·HCL or NHS addition (e5). The stretching vibrations of the amide I and amide II band in e4 are significantly stronger than those of e5, indicating the successful connection of ANG through the chemical amide reaction. (F) Hemolysis assessment of Lips‐encapsulating MEL or α‐MEL at different concentrations. 1% of Triton X‐100 was used as the positive controls. (G) C57BL/6 mice were administered with Lips (vehicle), RES‐Lips (RES), α‐MEL‐Lips (α‐MEL) or α‐MEL‐RES‐Lips (α‐MEL‐RES) via the tail vein for 14 days. Mice given PBS were used as the controls. The structure of heart, liver, spleen, lung, kidney and brain were examined by hematoxylin and eosin (H&E) staining. Scale bars: Heart, 5 μm; liver, kidney, spleen and lung, 50 μm; brain, 100 μm.

Given that hydrophilic drugs can be incorporated into the aqueous core of liposomes and that lipophilic agents can be loaded onto the phospholipid layer [42], we performed a three‐step procedure to synthesize α‐MEL‐RES‐Lips, which involved RES‐Lips formation, α‐MEL loading, and AGN coupling. The EE and LE were determined (Table S2). As shown in Figure 1A and Table S3, the average size of the liposome carriers (vehicle), RES‐Lips, and α‐MEL‐RES‐Lips were 67.9 ± 1.73 nm, 71.85 ± 0.08 nm, and 66.55 ± 2.42 nm, respectively, and the polydispersity indices (PDIs) were 0.11 ± 0.02, 0.11 ± 0.01, and 0.12 ± 0.02, respectively. The zeta potentials of the vehicle, RES‐Lips, and α‐MEL‐RES‐Lips were 1.33 ± 0.82 mV, 0.91 ± 0.15 mV, and 0.45 ± 0.13 mV, respectively (Figure 1B and Table S3). TEM confirmed that the delivery system exhibited a regular spherical shape (Figure 1C). The release of RES from the liposomes was evaluated at pH 6.8 and pH 7.4. A burst release followed by the sustained and complete release process was observed (Figure 1D). In the first 2 h, the release rate of RES in Lips was respectively 37.92% ± 1.91% at pH 6.8 and 23.48% ± 6.96% at pH 7.4, and 83.35% ± 2.84% (pH 6.8) and 68.93% ± 7.33% (pH 7.4) in the 24 h, and kept a sustained release behavior in the following time points. The stable releases of α‐MEL were as well (Figure S1). FT‐IR spectroscopy indicated that RES and α‐MEL were successfully incorporated in the liposomes (Figure 1E). Importantly, treatment with 0.5 μM free MEL resulted in complete RBC lysis. In contrast, α‐MEL‐Lips containing up to 10 μM MEL did not exhibit significant hemolysis (Figure 1F), demonstrating that α‐MEL‐RES‐Lips could significantly increase the hemocompatibility of MEL. To evaluate toxicity in vivo, the heart, liver, spleen, lung, kidney, and brain of each mouse were collected after treatment with vehicle, RES‐Lips, α‐MEL‐Lips, or α‐MEL‐RES‐Lips for 14 days via intravenous injection, and the tissue structures were examined by H&E staining. As shown in Figure 1G, none of the tissues in any of the groups presented with marked abnormalities. In addition, there were no significant differences in the physiological examination of blood cells (Figure S2) and biochemical test of serum (Figure S3) among groups.

### 3.2 Inhibitory Effects of α‐MEL‐, RES‐, and α‐MEL‐RES‐Lips on the Growth of GB Cells In Vitro

**FIGURE 2:**
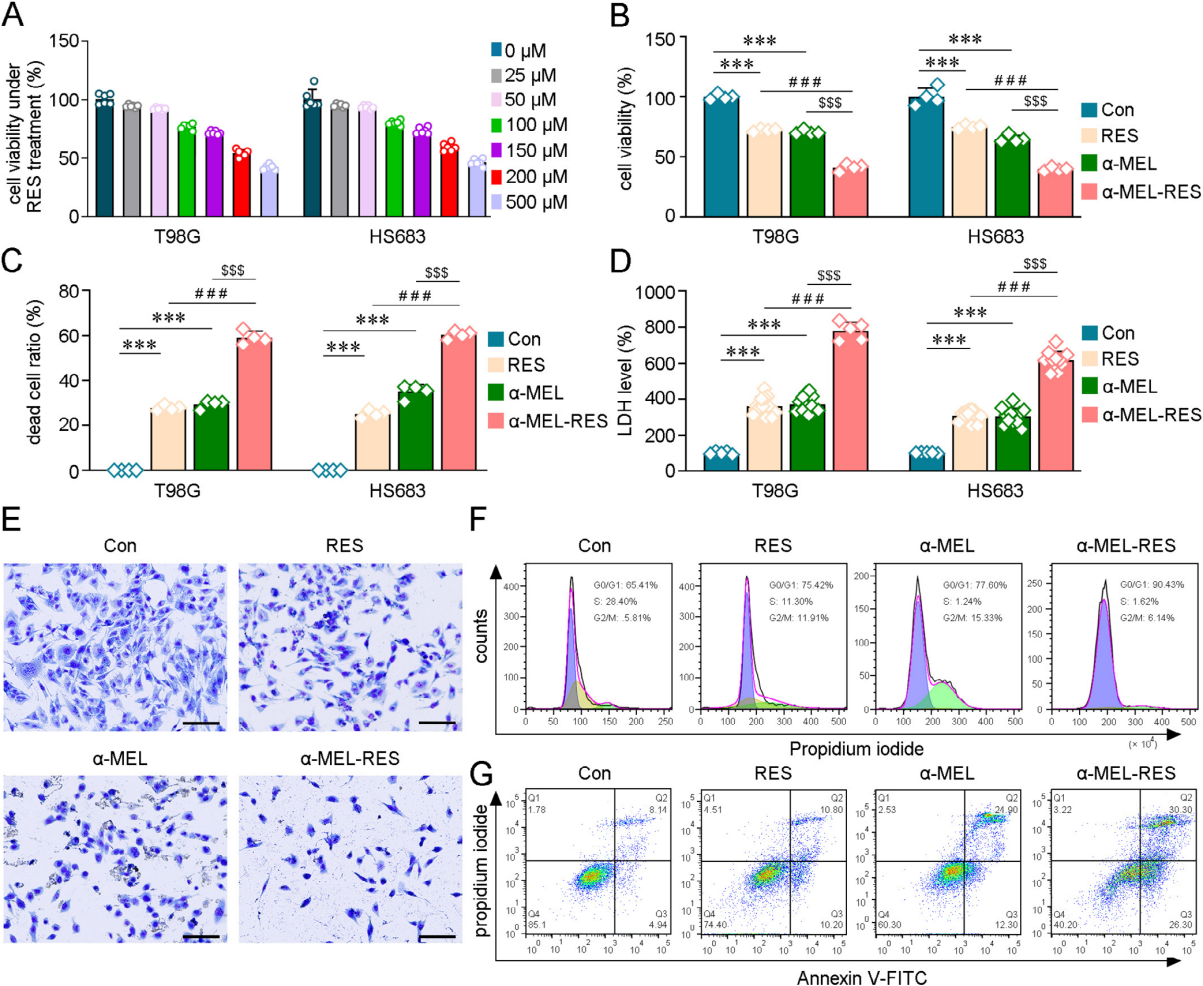
Inhibitory effects of RES‐, α‐MEL‐ and α‐MEL‐RES‐Lips treatment on GB cells. (A) The cell viability were assessed in the T98G and HS683 cells line by CCK‐8 after different concentrations of RES treatment for 24 h. *n* = 6. (B, C) Cell viability and dead ratio analysis showed that the combined treatment of α‐MEL (2 μM) with RES (150 μM) impeded the cells growth and increased the dead in the T98G and HS683 cells. Lips treatment was used as the vehicle control (Con). *n* = 4. (D) The release of lactic dehydrogenase (LDH) was evaluated. *n* = 5–12. (E) The alterations of morphology in the HS683 cells were examined by Giemsa staining. Scale bars: 50 μm. (F) Cells cycle were evaluated by flow cytometry (FCM) assays in the HS683 cells. (G) The apoptosis levels in the HS683 cells were determined by FCM analysis. All values represent mean ± SD, *n* = 6 in each group. ***p < 0.001 versus Con; ###*p* < 0.001 versus RES‐treated group; $$$*p* < 0.001 versus α‐MEL treatment group by one‐way analysis of variance (ANOVA) with post hoc Fisher's LSD.

The viability of GB cells was evaluated after RES‐Lips, α‐MEL‐Lips, and α‐MEL‐RES‐Lips treatment. As shown in Figure 2A, RES‐Lips reduced GB cell viability in a dose‐dependent manner in T98G (*F* [6, 35] = 459.248, *p* < 0.001) and HS683 cells (*F* (6, 35) = 164.053, *p* < 0.001). Treatment with 50 μM, 100 μM and 500 μM RES for 24 h led to decreases in the viability of T98G cells to 91.70% ± 1.13%, 76.89% ± 2.33%, and 41.73% ± 2.14% relative to that of the control, respectively, while HS683 cell viability was reduced to 93.09% ± 1.58%, 79.73% ± 2.08%, and 45.76% ± 2.97% compared with that of the control, respectively, as free RES‐mediated inhibition of GB cells (Figure S4A). α‐MEL‐Lips also exhibited inhibitory effects on T98G and HS683 cells in a dose‐dependent manner (Figure S4B). Treatment with α‐MEL‐RES‐Lips (containing 150 μM RES and 2 μM α‐MEL) for 24 h caused significant decreases in the cell viability to 41.00% ± 2.92% of controls in T98G (*F* [3, 12] = 496.63, *p* < 0.001) and 39.74% ± 1.69% of controls in HS683 cells (*F* [3, 12] = 140.40, *p* < 0.001; Figure 2B). The inhibitory effects of 150 μM of RES and 2 μM of α‐MEL in combination were shown to be synergistic (Combination index < 1) in both T98G and HS683 cell line as evaluated by CompuSyn software (Compusyn software for drug combinations for general dose‐effect analysis, and users guide combosyn unicorp, http://www.combosyn.com) (Figure S5). The results of the dead cell ratio confirmed those treatment‐mediated inhibitions (Figure 2C). LDH assays confirmed that α‐MEL and RES synergistically inhibited these cells (Figure 2D). Alterations in cell morphology were observed in HS683 cells after RES‐Lips, α‐MEL‐Lips, or α‐MEL‐RES‐Lips treatment. As shown in Figure 2E, the modified Giemsa staining results revealed that the GB cell bodies shriveled and that the protuberances were shorter after α‐MEL‐RES‐Lip treatment compared with vehicle controls. The destruction of cell morphology resulting from the combination of α‐MEL and RES was more significant than that resulting from RES or α‐MEL treatment alone. RES or α‐MEL treatment arrested HS683 cells in the G0/G1 phase and led to a decrease in the proportion of S‐phase cells (Figure 2F). The combination of α‐MEL and RES enhanced the above effects. Annexin V‐FITC/PI staining revealed that compared with RES‐Lips treatment, combined treatment with α‐MEL and RES led to more apoptotic HS683 cells (Figure 2G).

### 3.3 ANG‐Modified α‐MEL‐RES‐Lips Increased the GB‐Targeted Drug Delivery Across the BBB

**FIGURE 3:**
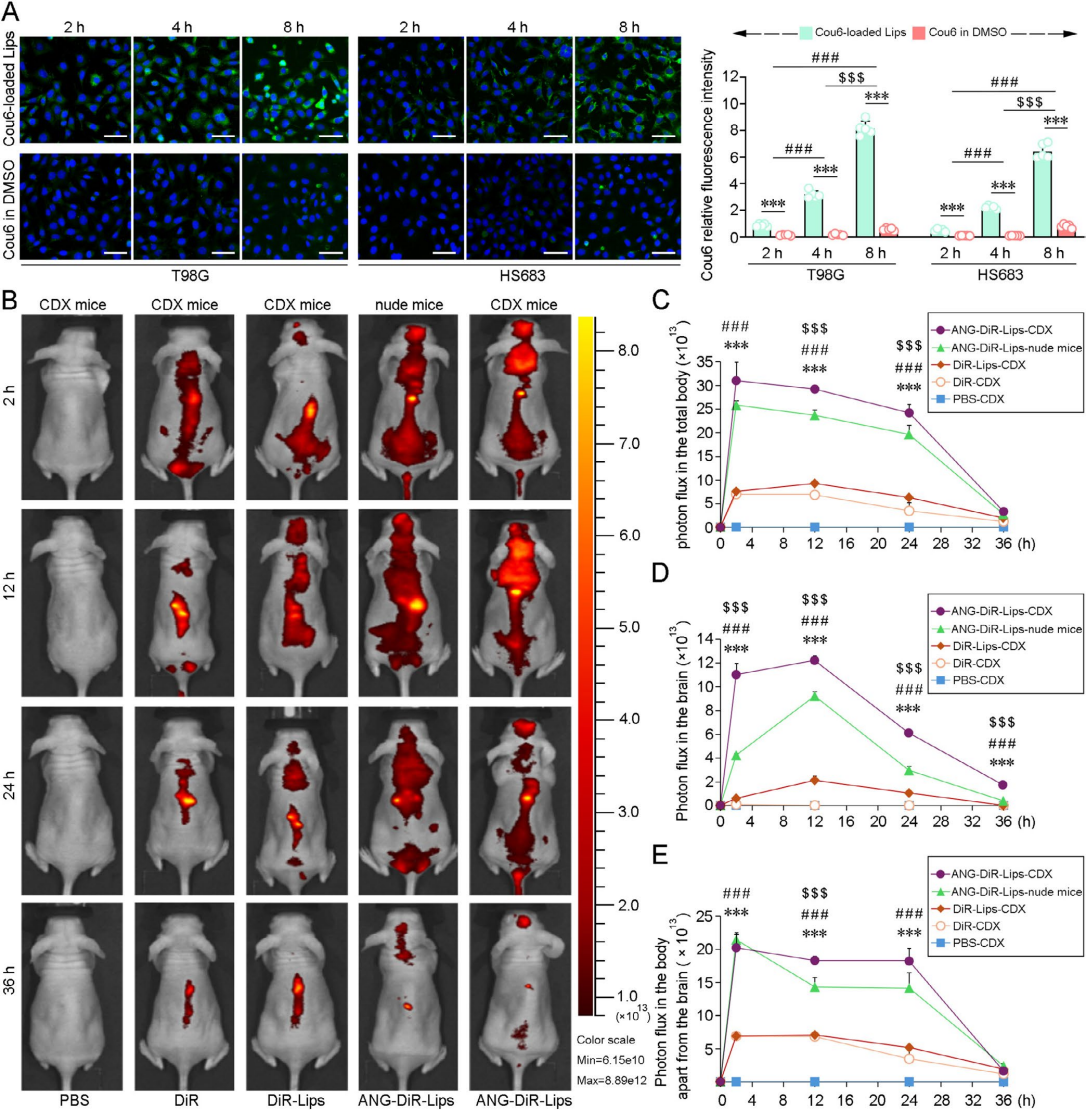
Assessment of the transport efficiency of ANG‐modified Lips across blood tumor barrier and blood tumor barrier. (A) A transwell assay in which hBMECs cells were planted in the upper chamber and T98G or HS683 cell were plated in the lower chamber was performed. After treated with DMSO‐dissolved Cou6 or ANG‐modified Lips encapsulating Cou6 (Cou6‐loaded Lips) in the upper chamber for 2 h, 4 h and 8 h, the green fluorescent signal of GB cells in the lower chamber was examined. Scale bar = 30 μm. All values represent mean ± SD, ***p < 0.001 versus DMSO‐dissolved Cou6 treatment group; ###*p* < 0.001 versus treatment for 2 h; $$$*p* < 0.001 versus treatment for 4 h. *n* = 5 independent biological replicates. (B) IVIS Spectrum assays were performed to assess the ability of ANG‐modified Lips to penetrate the blood brain barrier and blood tumor barrier in HS683 cell‐line‐derived xenograft (CDX) mice. The distribution of ANG‐modified Lips were traced by DiR at various time points. Quantification of photon flux in the total body (C), brain (D) and other part in the body except for the brain (E). All values represent mean ± SD, *n* = 5–6 in each group. ***p < 0.001 versus DiR‐treated CDX mice; ###*p* < 0.001 versus DiR‐Lips treatment CDX mice; $$$*p* < 0.001 versus nude mice given ANG‐modified Lips encapsulating DiR treatment by one‐way ANOVA with post hoc Fisher's LSD.

The ability of the nanosystem to penetrate the BBB was assessed in hBMECs via a transwell assay. Coumarin‐6 (Cou6) was loaded into the liposomes to trace the fluorescence signals. As shown in Figure 3A, the fluorescence signals in the T98G cells and HS683 cells treated with ANG‐modified liposomes were increased in a time‐dependent manner and BBB transport peaked at 8 h, whereas the fluorescence signal was not obviously detected in the cells treated by DMSO‐dissolved Cou6. The efficacy of ANG‐modified liposome delivery in vivo was analyzed by tracing DiR fluorescence after caudal vein injection. Body images of the mice were acquired with an in vivo imager (Figure 3B,C). Quantitative analysis revealed that the targeting efficiency was markedly greater in the brains of HS683‐luc cell xenograft (CDX) mice treated with ANG‐modified liposomes encapsulating DiR than in the brains of CDX mice treated with free DiR. No fluorescence signal was detected in the brains of free DiR‐treated CDX mice. The fluorescence distribution in the CDX mice treated with DiR‐liposomes was lower than that in the CDX mice treated with ANG‐DiR‐liposomes at 2, 12, 24, and 36 h after injection. In contrast, after ANG‐DiR‐liposome treatment, the fluorescence signals were stronger in the brains of the CDX mice than those of the nude mice without bearing xenografts, suggesting that the ANG modification led to efficient brain and tumor targeting.

### 3.4 ANG Modified α‐MEL‐RES‐Lips Exhibit Enhanced Anti‐GB Properties In Vivo

**FIGURE 4:**
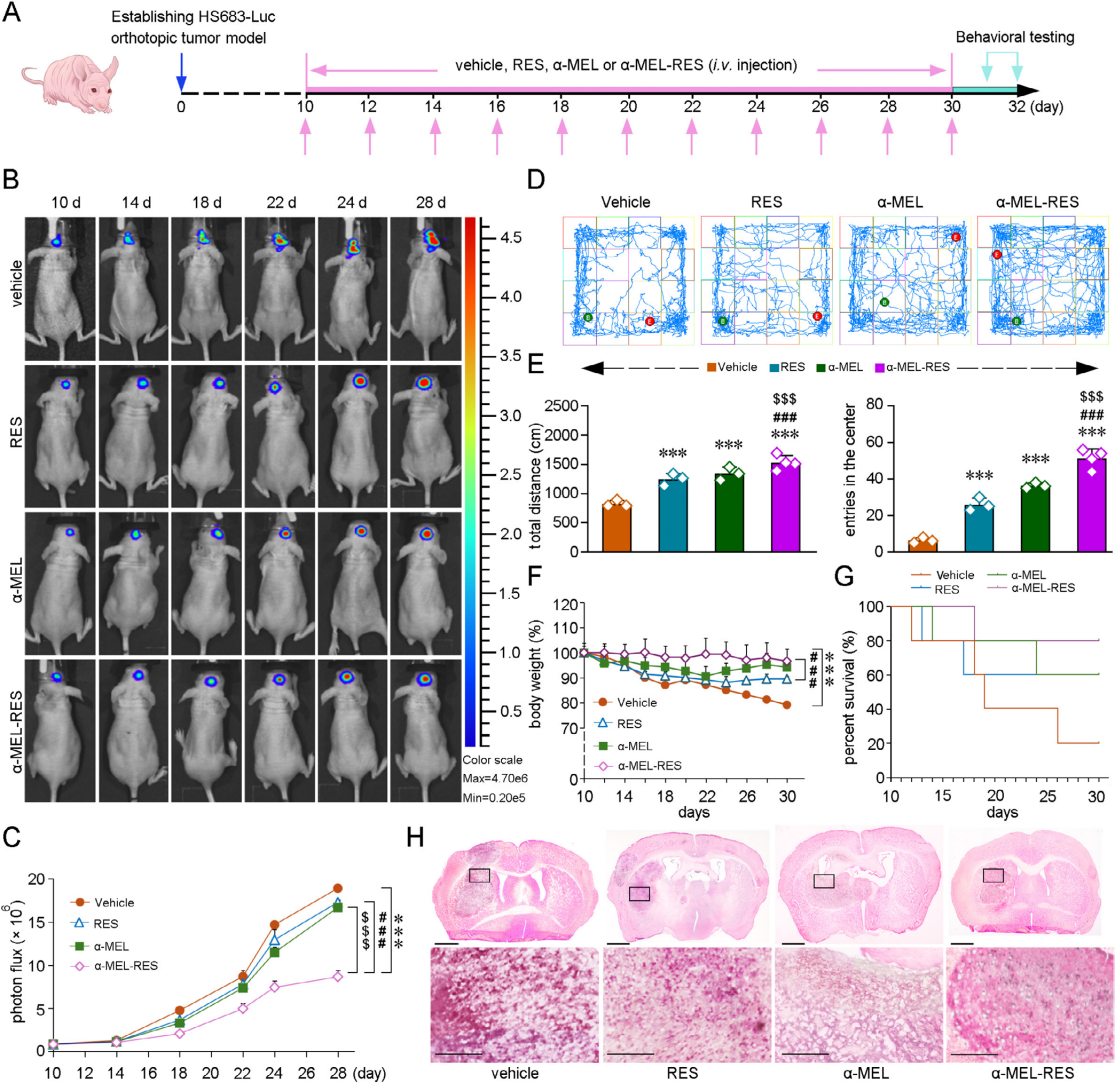
Combined treatment of α‐MEL with RES suppresses GB growth. (A) Experimental schedule. (B) Representative luminescence images showing the tumor in the brain of orthotopic HS683‐Luc glioblastoma‐bearing nude mice underwent the treatment of ANG‐modified RES‐Lips (RES), ANG‐modified α‐MEL‐Lips (α‐MEL) or ANG‐modified α‐MEL‐RES‐Lips (α‐MEL‐RES). (C) Quantification of the photon flux in the mice brains. (D) Representative traces showing the spontaneous motor and exploratory behavior of the mice in the open field test (OFT). (E) Quantification of the total distance and the number of entries into the center area that the mice performed in the OFT. *n* = 3–4. (F) The alterations of body weight in the mice. (G) Quantitative analysis of survival rates in the mice. (H) Hematoxylin and eosin (H&E) staining showing the tumor in the mice brains, scale bar = 1 mm. The high magnification images in bottom panels corresponding to the regions indicated in the boxes, scale bars = 250 μm. All values represent mean ± SD, *n* = 6–9 in each group. ***p < 0.001 versus vehicle‐treated group; ###*p* < 0.001 versus RES‐treated group; $$$*p* < 0.001 versus α‐MEL treatment group by one‐way ANOVA with post hoc Fisher's LSD.

The antitumor effects of ANG‐modified α‐MEL‐RES‐Lips were assessed in model mice bearing HS683‐luc orthotopic xenografts. A schematic of the treatment timeline is shown in Figure 4A. GB status was monitored by FT‐IR spectroscopy (Figure 4B). Compared with PBS‐treated xenograft mice, the mice treated with ANG‐modified RES‐Lips (RES), ANG‐modified α‐MEL‐Lips (α‐MEL) and ANG‐modified α‐MEL‐RES‐Lips (α‐MEL‐RES) exhibited delayed GB growth (Figure 4C). Importantly, α‐MEL‐RES produced the best effect. The neurological functions and locomotor abilities of the GB tumor‐bearing mice were investigated via the open field test (OFT). As shown in Figure 4D and Figure 4E, the total distance traveled to explore the field (*F* [3, 9] = 26.86, *p* < 0.001) and the number of entries into the center zone (*F* [3, 9] = 91.99, *p* < 0.001) performed by the GB mice were increased after RES, α‐MEL, and α‐MEL‐RES treatment compared to those given PBS treatment, and the combination of α‐MEL and RES exhibited synergistic effects. The body weights of the CDX mice declined with increasing tumor malignancy. The CDX mice treated with PBS lost weight more rapidly than the CDX mice administered RES, α‐MEL, and α‐MEL‐RES treatment groups; in contrast, the declines in the body weights of the α‐MEL‐RES‐treated CDX mice were moderate (Figure 4F). α‐MEL and RES treatment prolonged the lifespan of the CDX mice, and this phenomenon was more prominent in the α‐MEL‐RES‐treated GB CDX mice (Figure 4G). Brain sections selected at equal intervals were obtained (sections from bregma 1.7 mm to bregma −0.8 mm) for examining the area bearing xenograft tumor cells. Morphological analysis by H&E staining confirmed the antitumor effects of the interventions described above (Figure 4H). Dense areas of GB cells, which are characteristic of GB malignancies, were present in the vehicle‐treated xenograft mice. In contrast, the density of GB cells was markedly lower in the brains of the α‐MEL‐RES‐treated mice.

### 3.5 α‐MEL‐RES Treatment Promotes Apoptosis and Pyroptosis in GB Cells

**FIGURE 5:**
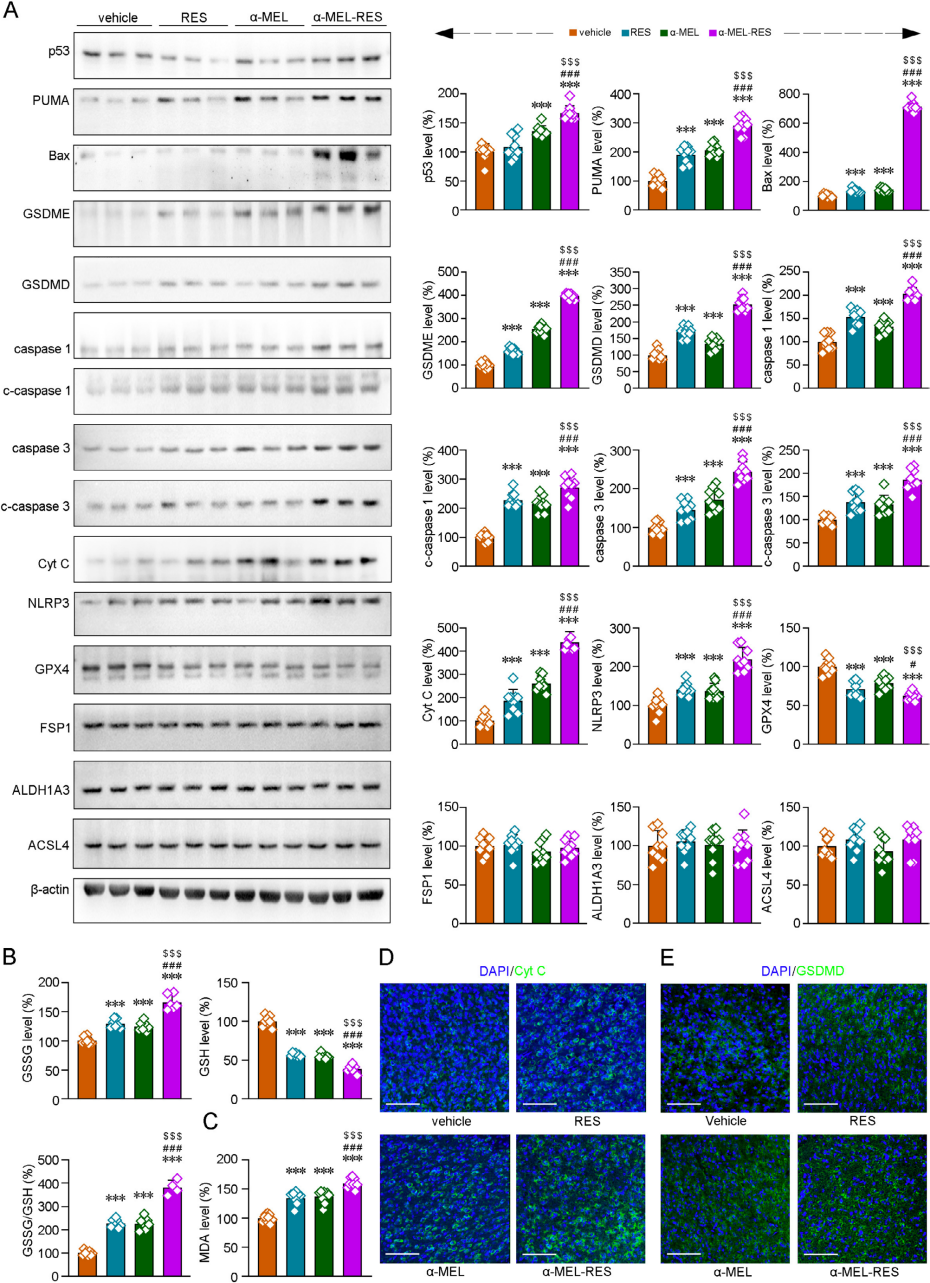
Effects of RES‐, α‐MEL‐ and α‐MEL‐RES management on the regulation of apoptosis and pyroptosis in GB cells. (A) Representative blots showing the expressions of apoptosis‐, pyroptosis‐ and ferroptosis‐related proteins in the HS683 cells. Quantitative analysis indicated that combined management of α‐MEL with RES increased the apoptosis and pyroptosis without significantly affecting the expressions of iron metabolism‐associated protein. (B) The levels of GSH, GSSG and the ratio of GSSG to GSH were measured to assess the oxidative‐redox status in the HS683 cells. (C) MDA contents were determined to evaluated the oxidation of lipids. Representative images showing the Cyt C (D) and GSDMD (E) protein levels in the tumor tissues of orthotopic HS683‐Luc glioblastoma‐bearing nude mice. 4′,6‐diamidino‐2‐phenylindole (DAPI) was used to label the nuclei. Scale bars: 60 μm. All values represent mean ± SD, *n* = 9 in each group. ***p < 0.001 versus vehicle‐treated group; #*p* < 0.05, ###*p* < 0.001 versus RES‐treated group; $$$*p* < 0.001 versus α‐MEL treatment group by one‐way ANOVA with post hoc Fisher's LSD.

Considering the potential of RES to induce pyroptosis and ferroptosis in human liver cancer cells and human neuroblastoma cells for therapeutic purposes [43], we investigated whether RES, α‐MEL or α‐MEL‐RES treatment could induce pyroptosis in GB cells. As shown in Figure 5A and Figure S6, the protein expressions of p53 (*F* [3, 32] = 38.59, *p* < 0.001), PUMA (*F* [3, 32] = 91.34, *p* < 0.001), Bax (*F* [3, 32] = 232.20, *p* < 0.001), gasdermin‐D (GSDMD) (*F* [3, 32] = 123.35, *p* < 0.001), gasdermin E (GSDME) (*F* [3, 32] = 850.52, *p* < 0.001), cleaved caspase 1 (c‐caspase 1) (*F* [3, 32] = 64.48, *p* < 0.001), caspase 1 (*F* [3, 32] = 70.87, *p* < 0.001), cleaved caspase 3 (c‐caspase 3) (*F* [3, 32] = 32.23, *p* < 0.001), caspase 3 (*F* [3, 32] = 54.75, *p* < 0.001), cytochrome C (Cyt C) (*F* [3, 32] = 120.02, *p* < 0.001), NLR family pyrin domain containing 3 (NLRP3) (*F* [3, 32] = 43.83, *p* < 0.001) were increased under RES or α‐MEL treatment in HS683 cells, whereas the protein levels of glutathione‐peroxidase 4 (GPX4) were significantly reduced (*F* [3, 32] = 31.61, *p* < 0.001). The combined management of α‐MEL with RES enhanced the alterations of the above proteins (*ps* < 0.001). The protein levels of acyl‐CoA‐synthetase long‐chain family number 4 (ACSL4) (*F* [3, 32] = 1.97, *p* = 0.138), ferroptosis suppressor protein 1 (FSP1) (*F* [3, 32] = 0.776, *p* = 0.516) and aldehyde dehydrogenase (ALDH) 1A3 (*F* [3, 32] = 0.193, *p* = 0.901) were not significantly changed under RES, α‐MEL or α‐MEL‐RES treatment. The contents of GSH and Malondialdehyde (MDA) were assessed by colorimetry. As shown in Figure 5B, the GSH content was significantly reduced in the GB cells after the RES and α‐MEL‐RES treatments (*F* [3, 24] = 206.96, *p* < 0.001), whereas the MDA contents were increased (*F* [3, 31] = 60.81, *p* < 0.001; Figure 5C). Immunofluorescence evaluations of Cyt C and GSDMD in the tumor tissues of orthotopic HS683‐Luc GB‐bearing nude mice confirmed that the expressions of these proteins were increased in GB cells after RES, α‐MEL or α‐MEL‐RES treatment (Figure 5D).

### 3.6 α‐MEL‐RES Inhibits GB by Regulating EMT via the Wnt/β‐Catenin Signaling Pathway

**FIGURE 6:**
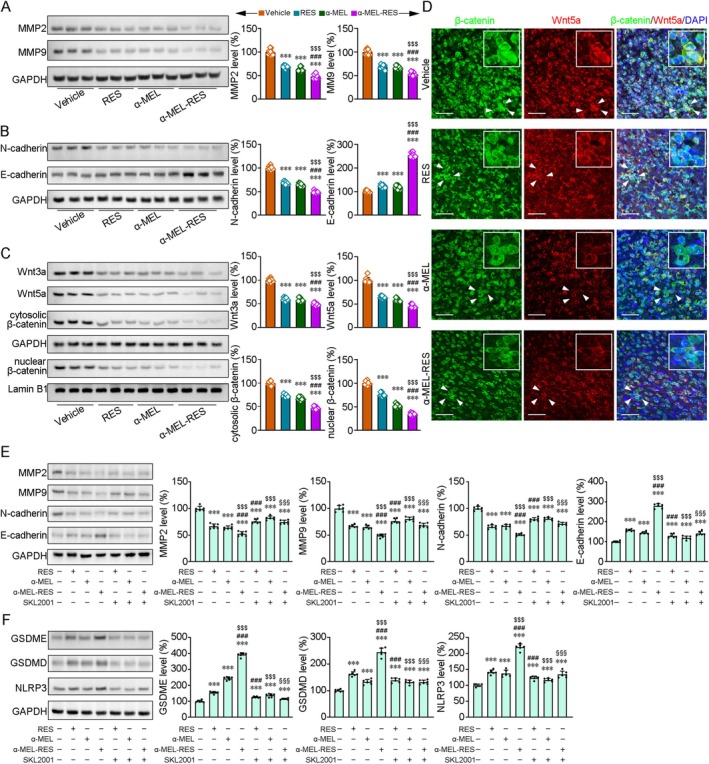
Combined management of liposomes co‐loaded with α‐MEL and RES enhanced the inhibition of EMT and induced the aggravation of pyroptosis via Wnt/β‐catenin signaling pathway in GB cells. (A) Representative blot showing the expressions of EMT‐realted proteins, MMP2 and MMP9, in the HS683 cells. (B) Western blot assays showing the protein levels of epithelial markers, E‐cadherin, and mesenchymal markers, N‐cadherin. (C) The protein expressions of Wnt3a, Wnt5a and β‐catenin were quantified. All values represent mean ± SD. ***p < 0.001 versus vehicle‐treated group; ###*p* < 0.001 versus RES‐treated group; $$$*p* < 0.001 versus α‐MEL treatment group by one‐way ANOVA with post hoc Fisher's LSD. *n* = 9 in each group. (D) Immunofluorescence staining showing the expressions of β‐catenin and Wnt5a in the tumor tissues of orthotopic HS683‐Luc glioblastoma‐bearing nude mice. The nuclei were visualized with DAPI. Scale bars: 50 μm. The white arrows indicate the enlarged image. (E) The alterations protein expressions of MMP2, MMP9, N‐cadherin and E‐cadherin triggered by RES, α‐MEL or α‐MEL‐RES treatment were abrogated by the treatment with the β‐catenin agonist, SKL2001 (40 μM, 24 h) in the HS683 cells. (F) The elevations of pyroptosis‐related protein expressions in the GSDME, GSDMD and NLRP3 were reversed by SKL2001 in the HS683 cells. ***p < 0.001 versus vehicle‐treated group; ###*p* < 0.001 versus RES‐treated group; $$$*p* < 0.001 versus α‐MEL treatment group; §§§*p* < 0.001 versus α‐MEL‐RES treatment group by one‐way ANOVA with post hoc Fisher's LSD. *n* = 6 in each group.

Activated epithelial–mesenchymal transition (EMT) signaling indicates the malignant growth and poor prognosis of GB [44]. Melittin can inhibit the growth of lung cancer [45] and colorectal cancer [46] cells by impeding EMT. Resveratrol suppresses EMT in both breast cancer cells [47] and GB cells [48]. Importantly, EMT acceleration can induce the transcriptional activation of GSDME for pyroptosis [49]. To investigate whether α‐MEL‐RES inhibits GB by regulating EMT, in the present study, we assessed the protein expressions of crucial EMT markers. As shown in Figure 6A and Figure S7, the protein levels of matrix metalloproteinase‐2 (MMP2) (*F* [3, 32] = 214.92, *p* < 0.001) and MMP9 (*F* [3, 32] = 181.99, *p* < 0.001) were reduced in the RES, α‐MEL, and α‐MEL‐RES treatment groups. After administration of RES, α‐MEL, or α‐MEL‐RES, the protein levels of E‐cadherin, an epithelial marker, were greater than those of vehicle controls in GB cells (*F* [3, 32] = 861.79, *p* < 0.001), whereas the protein expression levels of N‐cadherin, mesenchymal markers, were reduced (*F* [3, 32] = 404.13, *p* < 0.001; Figure 6B and Figure S7). Aberrant activation of Wnt/β‐catenin signaling could promote EMT to modulate GB pathology [50] by altering processes such as proliferation and motility [51]. In the present study, the protein expressions of Wnt3a (*F* [3, 32] = 363.53, *p* < 0.001), Wnt5a (*F* [3, 32] = 288.81, *p* < 0.001) and cytosolic β‐catenin were lower in the RES‐ and α‐MEL‐treated cells than in vehicle‐treated GB cells (*F* [3, 32] = 375.57, *p* < 0.001), and α‐MEL‐RES treatment showed greater efficiency (*ps* < 0.001; Figure 6C and Figure S7). RES‐, α‐MEL‐, and α‐MEL‐RES‐Lips induced decreases in nuclear β‐catenin as well (*F* [3, 32] = 608.28, *p* < 0.001). The results of immunofluorescence staining confirmed the RES‐ and α‐MEL‐ and α‐MEL‐RES‐mediated effects on the regulation of β‐catenin and Wnt5a in the tumor tissues of orthotopic HS683‐Luc GB‐bearing nude mice (Figure 6D). The α‐MEL‐, RES‐, and α‐MEL‐RES‐mediated suppression of EMT (Figure 6E) and pyroptosis (Figure 6F) was diminished by the Wnt/beta‐catenin pathway agonist SKL2001 (40 μM, 24 h) [52].

## 4 Discussion

Glioblastoma, a grade IV astrocytoma, is a devastating type of brain tumor characterized by malignant invasion, frequent recurrence, and a low response to treatment. Current therapeutic strategies mainly include surgery, chemotherapy, radiation, and immunotherapy, which are merely palliative; the median life expectancy of patients suffering from GB is only 1–1.5 years [1], and the five‐year survival rate of these patients is lower than 7% [53]. Thus, new effective therapeutic strategies are urgently needed. GB is not a single disease but rather a collection of diseases associated with different molecular changes due to genomic, transcriptomic, genetic, and epigenetic alterations. Disorders in signaling pathways, such as p53, Notch, Wnt, and nuclear factor‐κB pathways, might be related to the pathogenesis and progression of GB. Gousias and colleagues speculated that the formation of GB may be associated primarily with apoptosis escape [54]. EMT is a pivotal process in cancer progression. Tumor cells utilize EMT to increase their capacity to invade and metastasize. During EMT, cells lose their tight junctions, their apical–basal polarity is altered, and they are able to move, which may enhance cytoskeleton reorganization and increase the number of cells that break down the extracellular matrix, leading to the invasion of cancer cells into other tissues [55]. EMT is considered the critical factor of GB‐related signaling pathways. Among the regulators of EMT, Wnt signaling executes a variety of regulatory roles. Aberrant activation of Wnt signaling can lead to aggressive cell proliferation, adherence, self‐renewal, and differentiation and allow tumor cells to maintain their stemness properties [56]. Kahlert and colleagues [57] reported that activation of the Wnt/β‐catenin pathway caused the upregulation of Zeb1, an EMT‐activating transcription factor that is one of the most important components of classical EMT‐related cancer biology in GB cells. The increase in N‐cadherin protein expression is accompanied by the accumulation of β‐catenin [58]. The nuclear translocation of β‐catenin in carcinoma cells can upregulate EMT‐related genes such as N‐cadherin and vimentin. MMP‐2 and MMP9 are involved in the EMT. Reducing the expression of MMP‐2 proteins is related to the inhibition of proliferation, clonal growth, and metastasis, promoting the apoptosis of tumor cells [59]. Małek and colleagues observed melittin‐mediated inhibition of MMP2 and MMP9 in a dose‐dependent manner in GB cells [10]. In the present study, α‐MEL‐RES treatment increased the protein expression of E‐cadherin but reduced the protein levels of MMP2, MMP9, and N‐cadherin, suggesting that EMT was inhibited by α‐MEL‐RES.

Abnormal Wnt signaling may be responsible for the chemoresistance and radioresistance of GB cells [60]. Inhibiting the Wnt/β‐catenin pathway impeded tumor cell proliferation in a glioma model [61]. Resveratrol treatment impedes the proliferation and motility of highly malignant GB stem cells by reducing the protein expressions of β‐catenin and EMT activators [62]. It has been proposed that inhibiting Wnt signaling may be a possible therapeutic strategy for GB. Combination therapy has also been considered for GB treatment. At present, no drugs that target Wnt signaling pathways have been approved by the United States Food and Drug Administration. Suppressing the Wnt/β‐catenin pathway by combined therapy with celastrol and tumor necrosis factor‐related apoptosis‐induced ligand (TRAIL) synergistically suppressed the proliferation, migration, and invasion of GB cells and impeded EMT [63]. Resveratrol increases the sensitivity of glioma cells to temozolomide chemotherapy by suppressing Wnt signaling [64]. Resveratrol treatment inhibited the proliferation of glioma stem cells, significantly reduced their motility, and induced decreases in nuclear β‐catenin levels [62]. Melittin‐mediated suppression of the Wnt signaling pathway has been shown to reduce osteosarcoma lung metastasis [65] and impede human osteosarcoma in vitro [66], suggesting that resveratrol and melittin are potential agents to negatively regulate the Wnt signaling pathway and/or synergize for GB treatment. In the present study, α‐MEL‐RES treatment significantly reduced the protein expressions of Wn3a, Wnt5a, and β‐catenin in GB cells and in the tumor tissues of HS683 tumor‐bearing mice, indicating that α‐MEL‐RES suppressed GB.

Poor BBB permeability and resistance to multiple drugs are obstacles that restrict the therapeutic efficacy of small‐molecule anti‐GB agents [67]. As GB progresses, a blood–brain–tumor barrier forms; thus, chemotherapeutics must be more lipophilic, smaller, or administered at higher dosages, which might increase toxicity [68]. The low bioavailability of resveratrol and the hemolytic effects of melittin limit their use; therefore, how can the combination of resveratrol and melittin produce synergistic effects while reducing their side effects? Nanomaterials have been used to increase the solubility, stability, and effective concentration of resveratrol and to weaken the systemic toxicity of both melittin and resveratrol. Therefore, the development of a nanodelivery system comprising lipophilic structural components seems practical. Arcella and colleagues reported the brain‐targeting and tumor‐inhibiting effects of temozolomide‐loaded liposome biomolecular coronas [69]. In the present study, angiopep‐2‐modified liposomes were used to enhance brain targeting and tumor penetration. The liposomes were smaller than 100 nm and evenly distributed. RES release from the liposomes is at a pattern of initial burst release followed by the sustained and stable release process in an acidic environment, which is consistent with the release pattern of the liposomal drug carriers [70], indicating that the nanocarriers could overcome the acidic endosomal barrier. During the period of 24‐h treatment, both the free RES and the loaded RES may perform the effects in the body, which supported the notion that the toxicity and side effects of high‐dose drugs for anti‐tumor treatment should be paid attention. Nanoliposome‐encapsulating RES may attenuate the toxicity by controlling the release of the RES. Önay Uçar and colleagues observed that RES treatments could reduce the viability of glioma cells in a dose‐ and time‐dependent, while keeping normal cells alive. It has been reported that RES increases reactive oxygen species (ROS) production and leads to apoptosis of GB [27]. The combination of resveratrol with temozolomide could significantly increase the production of ROS in GB [71]. Resveratrol markedly caused the increases of ROS levels, growth arrest, and apoptosis in GB lines (U251) [72]. On the other hand, Wang and colleagues observed that RES may inhibit ROS generation, reducing ferroptosis of intestinal ischemia–reperfusion models in vitro [73]. RES could alleviate apoptosis and ROS production in a rat model of hyperoxia‐induced lung injury [74]. In the present study, treatments with RES‐ and α‐MEL‐RES‐Lips increased the contents of lipid peroxidation products, MDA, and elevated the ratio of GSSG to GSH, suggesting the increases of oxidative stress levels triggered by RES‐ and α‐MEL‐RES‐Lips. More in vitro and in vivo experiments may facilitate understanding RES for anti‐cancer effects and its application.

Considering the anti‐tumor effects of RES for glioma and its limitations of rapid metabolic processes and poor bioavailability, co‐administrations are attracting more attention for the strategies of enhancing phytochemical efficacy, especially when the approach is supported by nanotechnologies with targeting molecule modification. The combined management of RES with temozolomide caused cell cycle arrest, decreases of MMP9, and anti‐apoptosis protein Bcl‐2 in the human GB cell line (SHG44), and reduced the volume of the tumor in an orthotopic xenograft model of GB [71]. Hussain and colleagues observed that the paclitaxel‐resveratrol‐loaded Soluplus polymeric nanoparticles could enhance the bioavailability and anti‐glioma activity of the agents [75]. Kong and colleagues developed epirubicin‐resveratrol‐liposomes modified by p‐aminophenyl‐α‐D‐manno‐pyranoside and wheat germ agglutinin, which could improve BBB penetration and target brain tumor cells, and prolong the survival time of C6 glioma‐bearing rats [76]. Meanwhile, drug resistance is one of the major challenges for glioma treatment. Even with the combination of surgical resection and temozolomide treatments, patients suffering from glioma are still facing therapeutic resistance and a high recurrence rate. Interestingly, when binding onto the cell membrane, melittin can destabilize the phospholipid membrane of tumor cells and form pores, which may reduce the chance of melittin for drug resistance. The disrupted tumor cell membrane allows drugs to easily enter into the tumor cell, reducing drug efflux. Melittin increased the oxidant, anti‐tumor, and apoptotic effects of cisplatin in the GB cell line (DBTRG‐05MG) [11]. Combined administration of paclitaxel‐melittin‐loaded glycopeptide‐modified lipodisks exhibited synergistic effects against U87 human glioma cells and showed enhanced anti‐glioma impacts on glioma‐bearing mice [77]. In the present study, packaging α‐MEL in liposomes increases its hemocompatibility. ANG‐modified α‐MEL‐RES‐Lips increased the ability of the agents to cross the BBB, suppressing GB cell growth in vitro and in HS683 tumor‐bearing mice in vivo. α‐Melittin‐resveratrol treatment inhibited EMT by impeding the Wnt/β‐catenin signaling pathway.

Pyroptosis induced by the combination therapy of α‐MEL and RES may provide a potential approach for GB treatment. Pyroptosis is a type of programmed cell death related to inflammation. Like apoptosis, pyroptosis is accompanied by nuclear condensation and is involved in regulating tumor progression and response to therapeutics [78]. Wang and colleagues reported that chemotherapy drugs induced GSDME expression and could switch caspase‐3‐mediated apoptosis to pyroptosis [78]. Moreover, caspase family members induce the release of Cyt C, facilitating apoptosis cascade activation. Switching from apoptosis to pyroptosis results in good anti‐lung cancer efficiency [79]. The induction of apoptosis and pyroptosis could impede the development of drug resistance in non‐small cell lung cancer cells [80]. GSDME‐mediated pyroptosis suppresses head and neck cancer [81]. In the present study, α‐MEL, the melittin conjugated with an amphiphilic α‐helical peptide at its N‐terminus, induced apoptosis, which was associated with the activation of caspase 3 [11]. The inhibition of the cancer‐impeded function of p53 (encoded by *TP53*) is critical for tumor development in humans. p53 maintains unmutated in most cases of GB [82]. The inactivation of WT‐p53 in GB may promote tumor progression [83]. Considering the p53 mutations in T98G cells [84], the HS683 cell line was selected for the mechanism analysis of RES‐, α‐MEL‐, and α‐MEL‐RES‐mediated anti‐GB effects in the present study. RES‐, α‐MEL‐ and α‐MEL‐RES‐administration caused increases in the protein expressions of p53, PUMA (a modulator of apoptosis upregulated by p53) and Bax, which might facilitate HS683 GB cells apoptosis. RES‐, α‐MEL‐ and α‐MEL‐RES‐management upregulated the protein expressions of GSDMD and GSDME, enhancing the release of Cyt C. Importantly, combined treatment of α‐MEL with RES increased the protein expressions of cleaved‐caspase 3, which may initiate the switch from apoptosis to pyroptosis. On the other hand, Wu and colleagues reported that the prenylated chalcone isobavachalcone induced apoptosis in GB cells while mitigating pyroptosis [85]. The limitations of the present study include that the mechanism analysis of anti‐GB effects triggered by RES‐, α‐MEL‐, and α‐MEL‐RES treatments only identified protein expressions and revealed the changes related to apoptosis, pyroptosis, and Wnt/β‐catenin signaling pathway; whereas, the alterations of protein levels could not directly reflect gene changes. The anti‐tumor effects caused by the above agents on the GB with p53 mutation were not evaluated. Further study will allow for understanding and targeting apoptosis, pyroptosis, and the apoptosis‐pyroptosis switch in anti‐GB therapies.

In summary, the combination of α‐MEL with RES impedes GB cells by triggering apoptosis and pyroptosis. ANG‐modified liposomes encapsulating α‐MEL and RES increase BBB penetration and tumor cell selectivity. The distributions of the nanocarrier ANG‐modified Lips were significantly increased in the tumor tissue. Lips‐packaging inhibits α‐MEL‐mediated hemolytic activity. Furthermore, α‐MEL combined with RES dramatically increased the protein levels of MMP2, MMP9, and E‐cadherin but reduced the protein expression of N‐cadherin in GB cells, suggesting that the combined management of α‐MEL with RES synergistically regulates EMT. We observed that the α‐MEL‐RES‐caused induction of pyroptosis and EMT inhibition via the Wnt/β‐Catenin signaling pathway, thereby suppressing GB growth and prolonging the lifespan of GB tumor‐bearing mice. The results of the present study suggest that combination therapy of α‐MEL with RES may be a potential strategy for GB treatment.

## Author Contributions

W.L. designed the study. H.Q.Z., Y.W., X.G., M.D., Z.L., K.Y., Y.X., and N.X. performed the experiments. H.Q.Z., N.X., and Y.W. analyzed the data. H.Q.Z., Y.X., and N.X. wrote the manuscript.

## Conflicts of Interest

The authors declare no conflicts of interest.

## Supporting information


Figure S1.

Figure S2.

Figure S3.

Figure S4.

Figure S5.

Figure S6.

Figure S7.

Table S1.

Table S2.

Table S3.

## Data Availability

Data sharing not applicable to this article as no datasets were generated or analysed during the current study.
