# Supplementary figures and images for: ANG‐Modified Liposomes Coloaded With α‐Melittin and Resveratrol Induce Apoptosis and Pyroptosis in Glioblastoma Cells by Impeding Wnt/β‐Catenin Signaling
Source: CNS Neurosci Ther. 2025 May 21;31(5):e70437. doi: 10.1111/cns.70437 (PMC12095925; doi:10.1111/cns.70437)

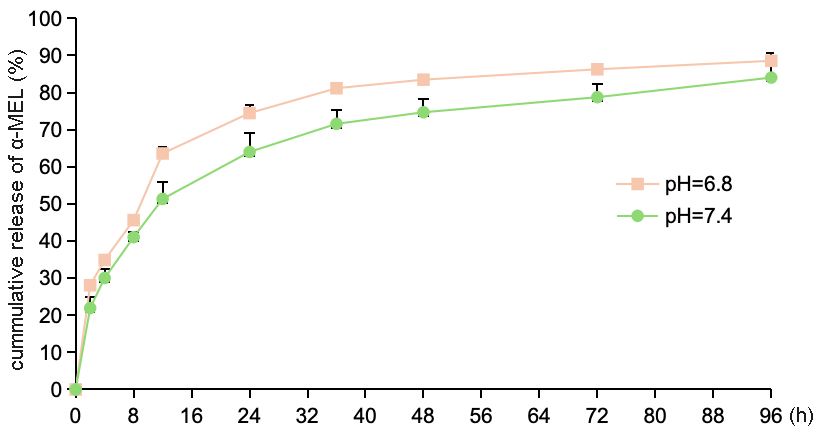

Supplement: Supplementary file 1 — Figure S1. [file CNS-31-e70437-s009.tif]

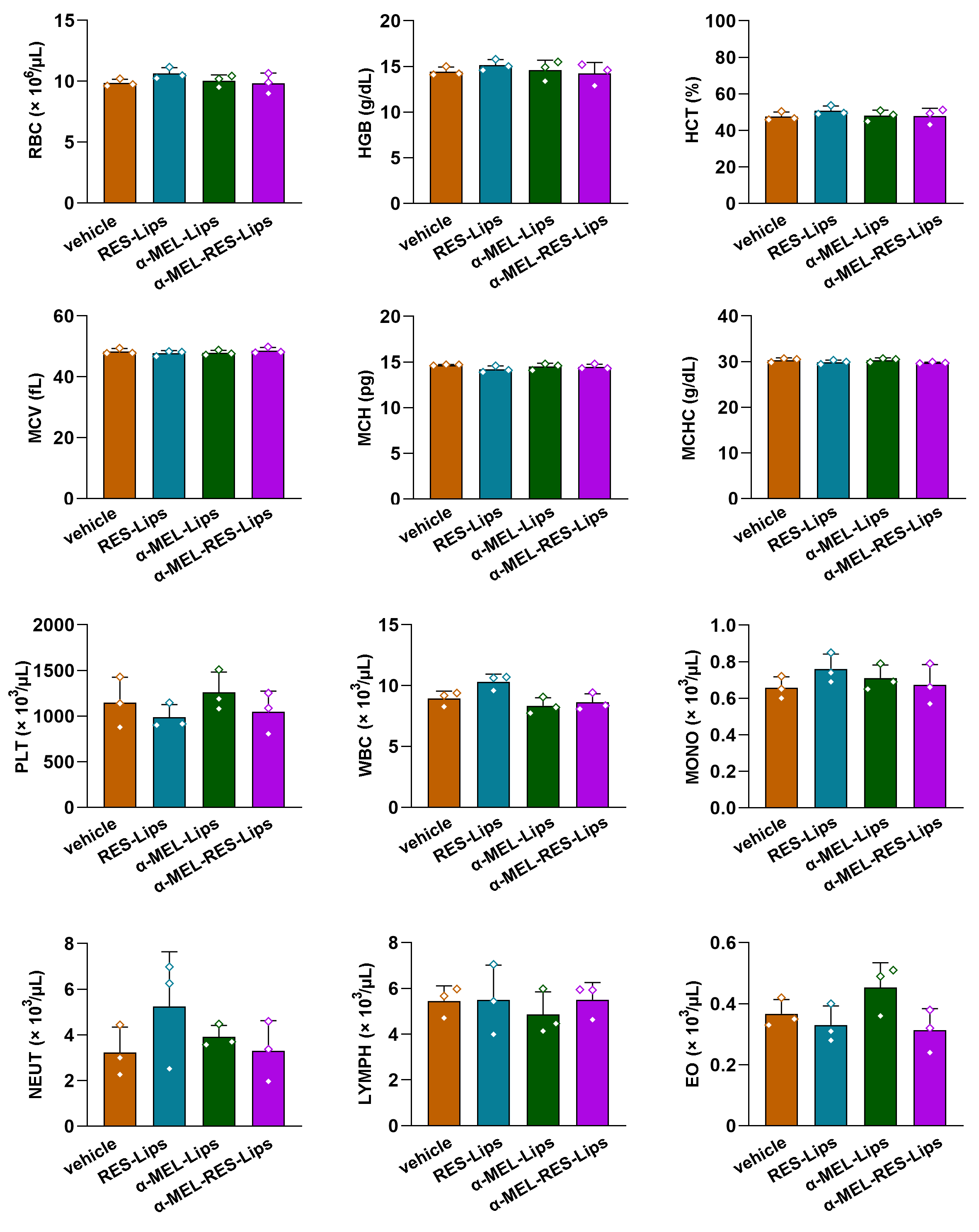

Supplement: Supplementary file 2 — Figure S2. [file CNS-31-e70437-s001.tif]

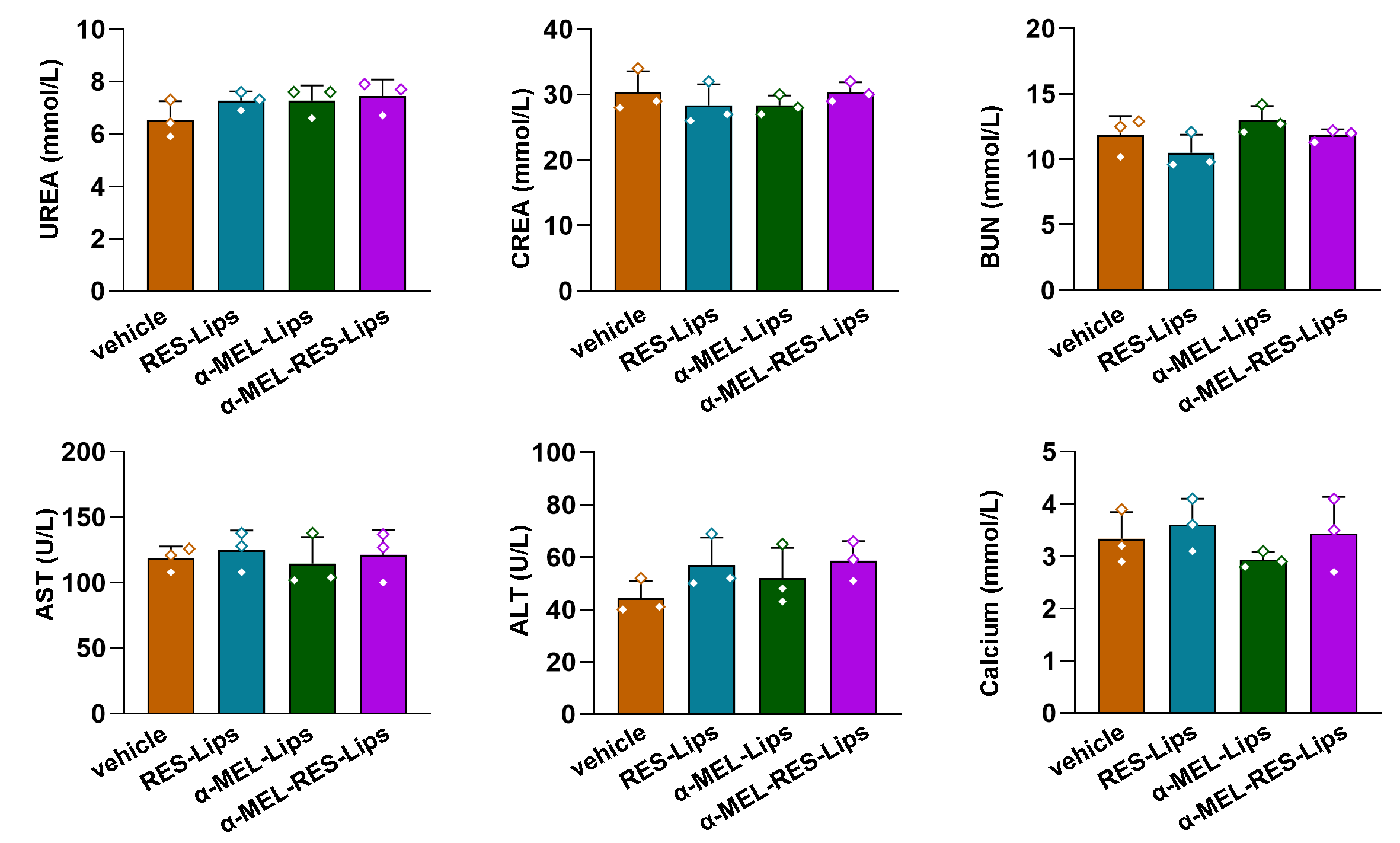

Supplement: Supplementary file 3 — Figure S3. [file CNS-31-e70437-s008.tif]

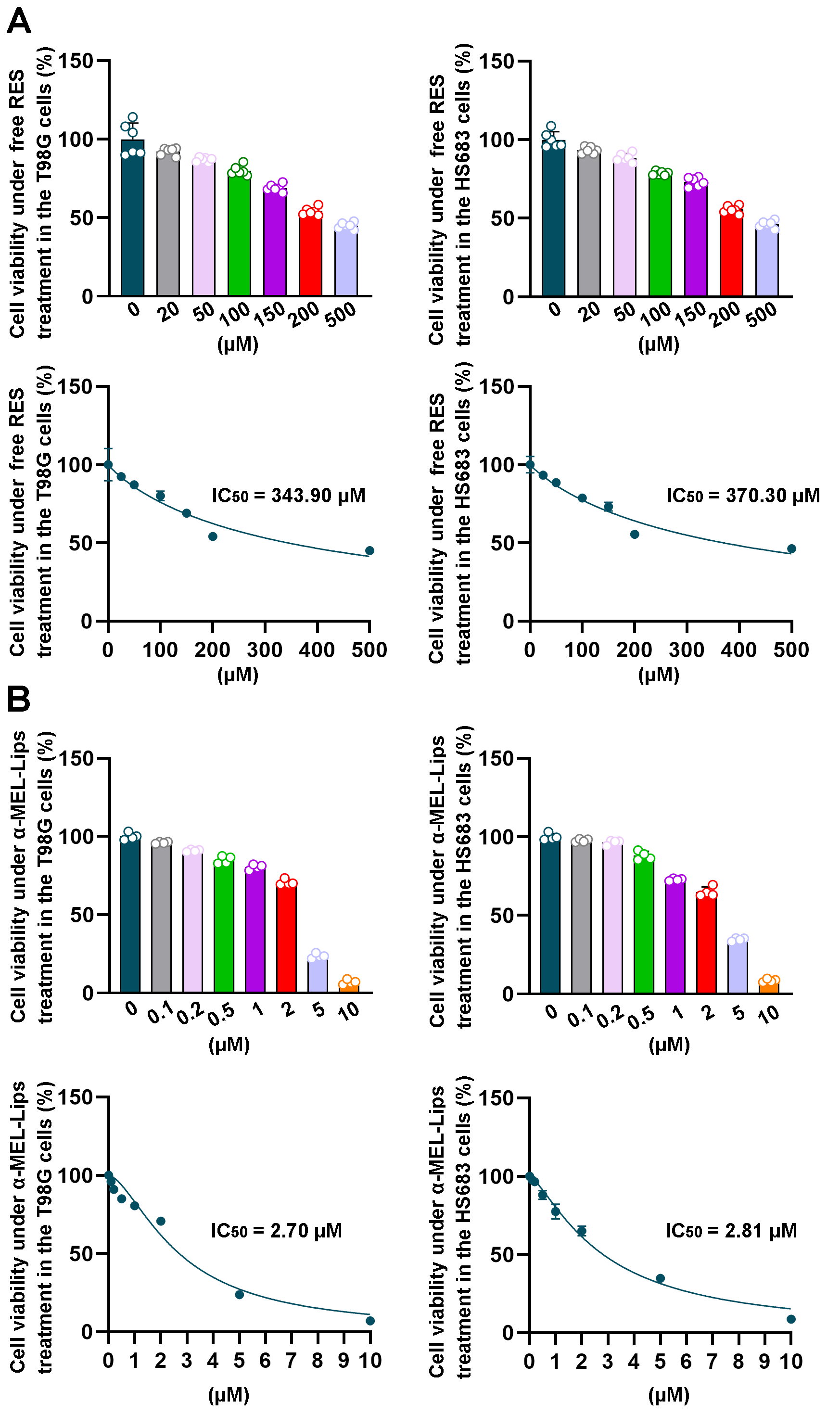

Supplement: Supplementary file 4 — Figure S4. [file CNS-31-e70437-s004.tif]

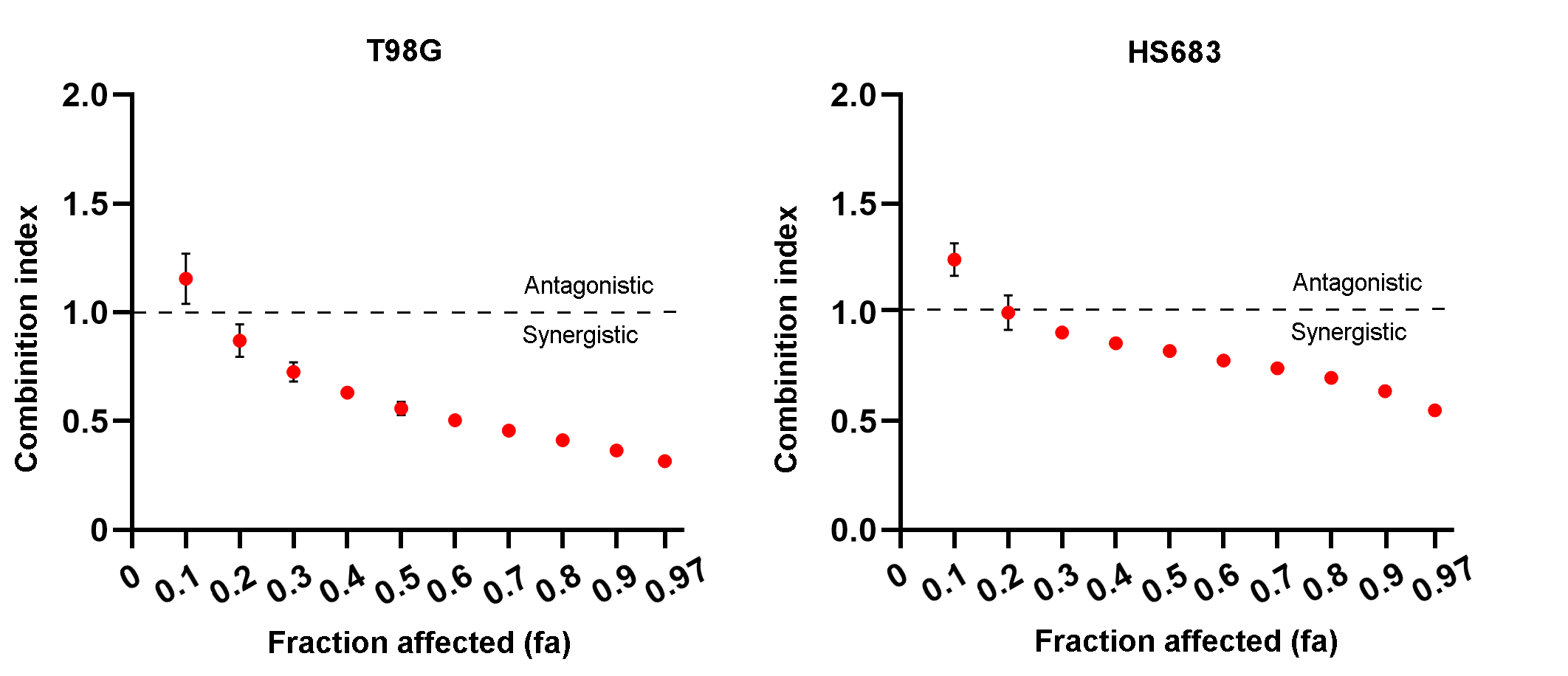

Supplement: Supplementary file 5 — Figure S5. [file CNS-31-e70437-s003.tif]

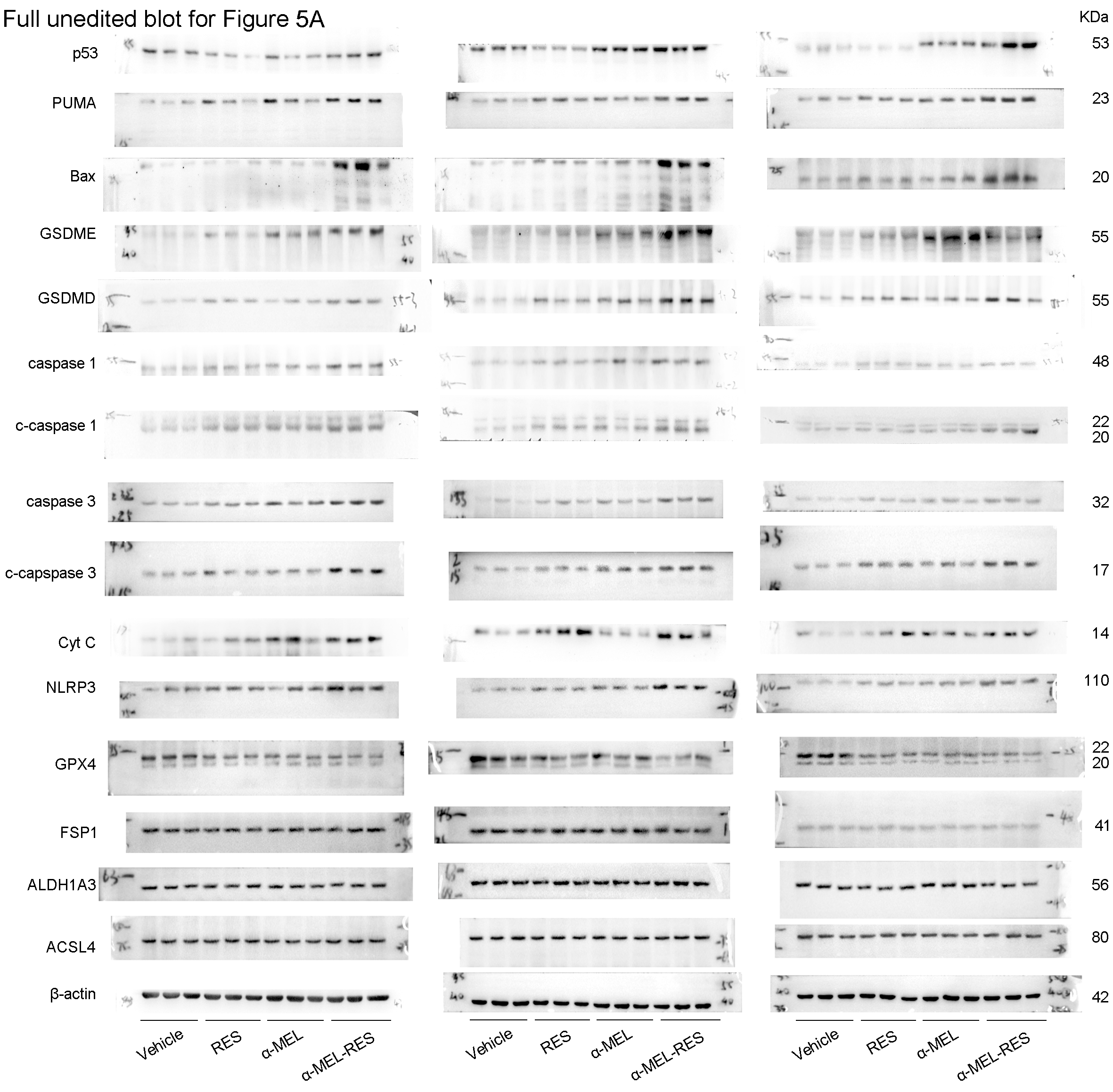

Supplement: Supplementary file 6 — Figure S6. [file CNS-31-e70437-s002.tif]

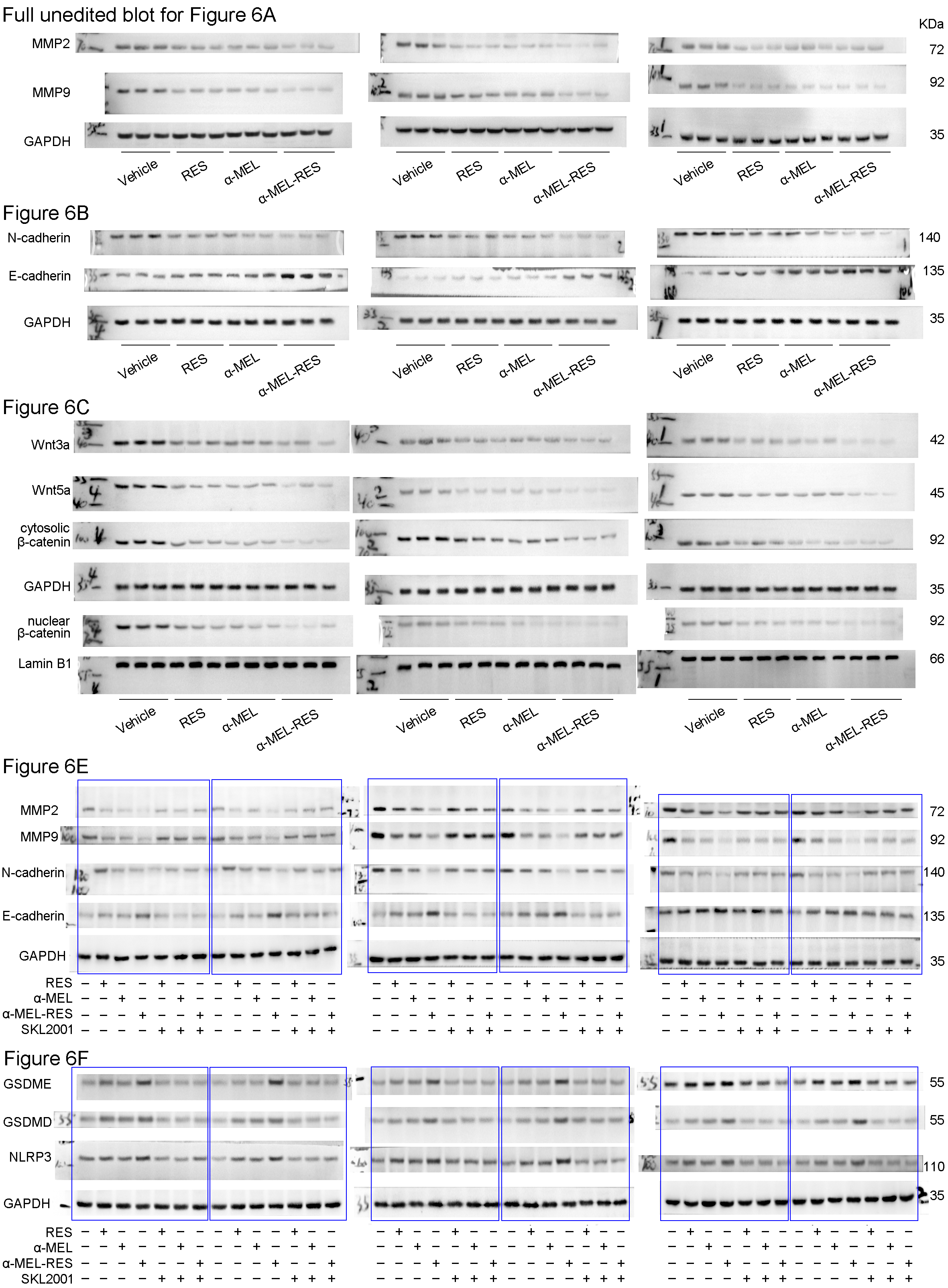

Supplement: Supplementary file 7 — Figure S7. [file CNS-31-e70437-s010.tif]
