# Supplementary material for: ANG‐Modified Liposomes Coloaded With α‐Melittin and Resveratrol Induce Apoptosis and Pyroptosis in Glioblastoma Cells by Impeding Wnt/β‐Catenin Signaling
Source: CNS Neurosci Ther. 2025 May 21;31(5):e70437. doi: 10.1111/cns.70437 (PMC12095925; doi:10.1111/cns.70437)
Supplement: Supplementary file 8 — Table S1. [file CNS-31-e70437-s006.docx]

**Supplementary Table S1 KEY RESOURCES**

| **REAGENT or RESOURCE** | **SOURCE** | **IDENTIFIER** | |
| --- | --- | --- | --- |
| Antibodies | Supplier | Catalogue number | Molecular Weight (kDa) |
| Rabbit anti-ACSL4 | Cell Signaling TECHNOLOGY | 38493 | 80 |
| Rabbit anti-ALDH1A3 | abcam | ab129815 | 56 |
| Mouse anti-β-actin | abcam | ab8226 | 42 |
| Mouse anti-β-catenin | absin | abs149656 | 92 |
| Rabbit anti-Bax | Cell Signaling TECHNOLOGY | 5023 | 20 |
| Rabbit anti-caspase 1 | Cell Signaling TECHNOLOGY | 3866 | 48 |
| Rabbit anti-caspase 3 | abcam | ab32351 | 32 |
| Rabbit anti-cleaved caspase 1 | Cell Signaling TECHNOLOGY | 4199 | 20, 22 |
| Rabbit anti-cleaved caspase 3 | Cell Signaling TECHNOLOGY | 9662 | 17 |
| Rabbit anti-Cytochrome c | Cell Signaling TECHNOLOGY | 11940 | 14 |
| Rabbit anti-E-cadherin | Cell Signaling TECHNOLOGY | 3195 | 135 |
| Rabbit anti-FSP1 | Cell Signaling TECHNOLOGY | 24972 | 41 |
| Mouse anti-GAPDH | Aksomics | KC-5G5 | 35 |
| Rabbit anti-GPX4 | Cell Signaling TECHNOLOGY | 52455 | 20, 22 |
| Rabbit anti-GSDMD | Cell Signaling TECHNOLOGY | 69469S | 55 |
| Rabbit anti-GSDME | absin | abs159339 | 55 |
| Mouse anti-MMP2 | absin | abs112854 | 72 |
| Mouse anti-MMP9 | absin | abs100167 | 92 |
| Rabbit anti-N-cadherin | Cell Signaling TECHNOLOGY | 13116 | 140 |
| Rabbit anti-NLRP3 | Cell Signaling TECHNOLOGY | 15101 | 110 |
| Rabbit anti-p53 | Cell Signaling TECHNOLOGY | 9282 | 53 |
| Rabbit anti-PUMA | Cell Signaling TECHNOLOGY | 4976 | 23 |
| **Critical Commercial Assays** | **Supplier** | **Catalogue number** |  |
| Annexin V-FITC Apoptosis Detection Kit | Cell Signaling Technology | 6592 |  |
| CATALYST UPC RATIO | IDEXX Laboratories | 98-11008-01 |  |
| CATALYST ALT | IDEXX Laboratories | 98-11067-01 |  |
| CATALYST AST | IDEXX Laboratories | 98-11069-01 |  |
| CATALYST BUN | IDEXX Laboratories | 98-11070-01 |  |
| CATALYST CREA | IDEXX Laboratories | 98-11074-01 |  |
| Cell Cycle Detection Kit | absin | abs50005 |  |
| MDA Detection Kit | Nanjing Jiancheng Bioengineering Institute | A003-1-2 |  |
| Nuclear Extraction Kit | Abcam | ab113474 |  |
| Toal glutathione/Oxidized glutathione assay kit | Beyotime Institute of biotechnology | A061-2-1 |  |
