# Supplementary material for: ANG‐Modified Liposomes Coloaded With α‐Melittin and Resveratrol Induce Apoptosis and Pyroptosis in Glioblastoma Cells by Impeding Wnt/β‐Catenin Signaling
Source: CNS Neurosci Ther. 2025 May 21;31(5):e70437. doi: 10.1111/cns.70437 (PMC12095925; doi:10.1111/cns.70437)
Supplement: Supplementary file 9 — Table S2. [file CNS-31-e70437-s007.docx]

**Supplementary Table S2** **The encapsulation efficiency and loading efficiency of RES-Lips**

| **Item** | **RES-Lips** |
| --- | --- |
| Encapsulation efficiency | 80.28 ± 2.67% |
| Loading efficiency | 8.82 ± 0.17% |
