# Supplementary material for: ANG‐Modified Liposomes Coloaded With α‐Melittin and Resveratrol Induce Apoptosis and Pyroptosis in Glioblastoma Cells by Impeding Wnt/β‐Catenin Signaling
Source: CNS Neurosci Ther. 2025 May 21;31(5):e70437. doi: 10.1111/cns.70437 (PMC12095925; doi:10.1111/cns.70437)
Supplement: Supplementary file 10 — Table S3. [file CNS-31-e70437-s005.docx]

**Supplementary Table S3** **The characteristics of the Vehicle, RES-Lips and α-MEL-RES-Lips**

| Sample | Size (nm) | Zeta potential (mV) | PDI |
| --- | --- | --- | --- |
| Vehicle | 67.9 ± 1.73 | 1.13 ± 0.82 | 0.11 ± 0.02 |
| RES-Lips | 71.85 ± 0.08 | 0.91 ± 0.15 | 0.11 ± 0.01 |
| α-MEL-RES-Lips | 66.55 ± 2.42 | 0.45 ± 0.13 | 0.12 ± 0.02 |
